# Pathways to cost competitive and viable lithium production from Salton Sea geothermal brines

**DOI:** 10.1038/s41467-026-75389-8

**Published:** 2026-07-09

**Authors:** Jannis Wesselkaemper, Theo Renaud, Naod Araya, Ken Dekkers, Joris Popineau, Jeremy Riffault, John O’Sullivan, Andrew Z. Haddad

**Affiliations:** 1 Energy Analysis and Environmental Impacts Division, Energy Technologies Area, Lawrence Berkeley National Laboratory, Berkeley, CA USA; 2 Geothermal Institute, University of Auckland, Auckland, New Zealand; 3 Energy Technologies and Systems Division, Lawrence Berkeley National Laboratory, Berkeley, CA USA

**Keywords:** Geothermal energy, Geochemistry, Energy economics, Policy

## Abstract

Lithium supply chains remain heavily concentrated in hard rock and brine resources, creating significant supply risks. Geothermal brines represent an underutilized alternative, yet commercial progress is hindered by the absence of facility-scale cost assessments. Here, we present a techno-economic analysis of large-scale lithium extraction from Salton Sea geothermal brines, drawing on primary company disclosures, process patents, and brine resource modeling. Caused by varying lithium and impurity concentrations, brine dilution over time, and process configurations (e.g., production via carbonation and conversion vs. electrolysis), we find that large-scale production costs may reach ~10,000 United States dollars per ton, but increase up to 22,000 United States dollars per ton with higher certainty of brine modeling, raising concerns about economic competitiveness to conventional low-cost sources. Finally, a project feasibility-focused scenario analyses shows that leveraging brine pre-treatment by-product sales could lower long-term lithium break-even prices by ~5,000 United States dollars per ton, whereas capital cost optimization of 20% could further reduce lithium break-even prices by 10%.

## Abstract (web summary)

Researchers find that producing lithium from California’s Salton Sea geothermal brines costs ~$10,000–22,000 per ton, exceeding current prices. By-product sales and capital subsidies could cut break-even prices enough to reach viability.

## Introduction

Lithium is a key material in current and future battery technologies used in electric vehicles, stationary energy storage, or portable electronics^1^. Global demand reached 165 kilo-tonnes per annum (kt a^−1^) in 2023, and is expected to rise to ~1300 kt a^−1^ by 2040^2^, intensifying efforts by governments and industry to secure reliable supply^3^. Primary lithium production has expanded by 650% over the past decade, going from 31.7 kt in 2014 to 240 kt in 2024^4^, but remains highly concentrated. Australia, China, Chile, Zimbabwe, and Argentina account for more than 90% of global mined supply^2,4^, while China refines 57% of this^2,5^. Moreover, production assets are largely controlled by a small number of companies, predominantly Chinese (e.g., Ganfeng and Tianqi) and U.S. companies (Albemarle), each holding roughly one-third of global mining capacity^6,7^.

These supply chain characteristics, combined with rising demand, render lithium a material of high criticality for regions such as Europe and the United States^3^. Although commercial supply currently comes exclusively from hard-rock deposits and continental brines^8^, lithium resources are globally distributed, creating opportunities for diversification alongside expanding battery recycling efforts^9–13^. Clay-hosted deposits in the United States, Mexico, China, and Serbia illustrate this potential and are attracting governmental support^8^, though recent studies emphasize that processing technologies must improve to compete internationally^8^, particularly under the current low-price environment (~10,000 USD per lithium carbonate equivalents (LCE)), down sharply from highs exceeding 80,000 United States dollars per ton (USD t^−1^) LCE in 2022^14^.

Geothermal brines represent another promising but under-developed resource^15,16^. Brines in the German Upper Rhine Valley and the Californian Salton Sea region contain elevated lithium concentrations of 160–210 and 200 mg L^−1^, respectively^17,18^, attracting increasing commercial and governmental interest^19^, despite being less concentrated than South American Salar brines (up to 2500 mg L^−1^). Initial techno-economic assessments suggest that operating costs for extracting and refining lithium from geothermal brines could be cost-competitive with conventional sources, with costs around 4000 USD t^−1^ LCE^20^. However, developing large-scale geothermal-based lithium production requires a comprehensive understanding of full project costs, including capital expenditures (CapEx), operating expenditures (OpEx), and sustaining capital (SusEx), as well as clear identification of optimization pathways necessary for long-term viability in a price-volatile market dominated by few influential actors.

In this study, we conduct a facility-scale TEA of lithium production from Geothermal Brines in California’s Salton Sea region. Using primary data on operational lithium concentration dilution, process technology patents, and company disclosures, we estimate baseline lithium production costs across operational scenarios, brine concentrations, and process configurations. We then identify key cost drivers spanning CapEx, OpEx, and SusEx and determine break-even lithium prices and financial viability under these baseline conditions. Finally, we evaluate scenario-based pathways, such as capital-cost reductions, by-product valorization, and process optimization, to improve economic competitiveness and increase the robustness of geothermal lithium production in volatile market conditions.

## Results

### Baseline facility-scale lithium production costs

**Fig. 1:**
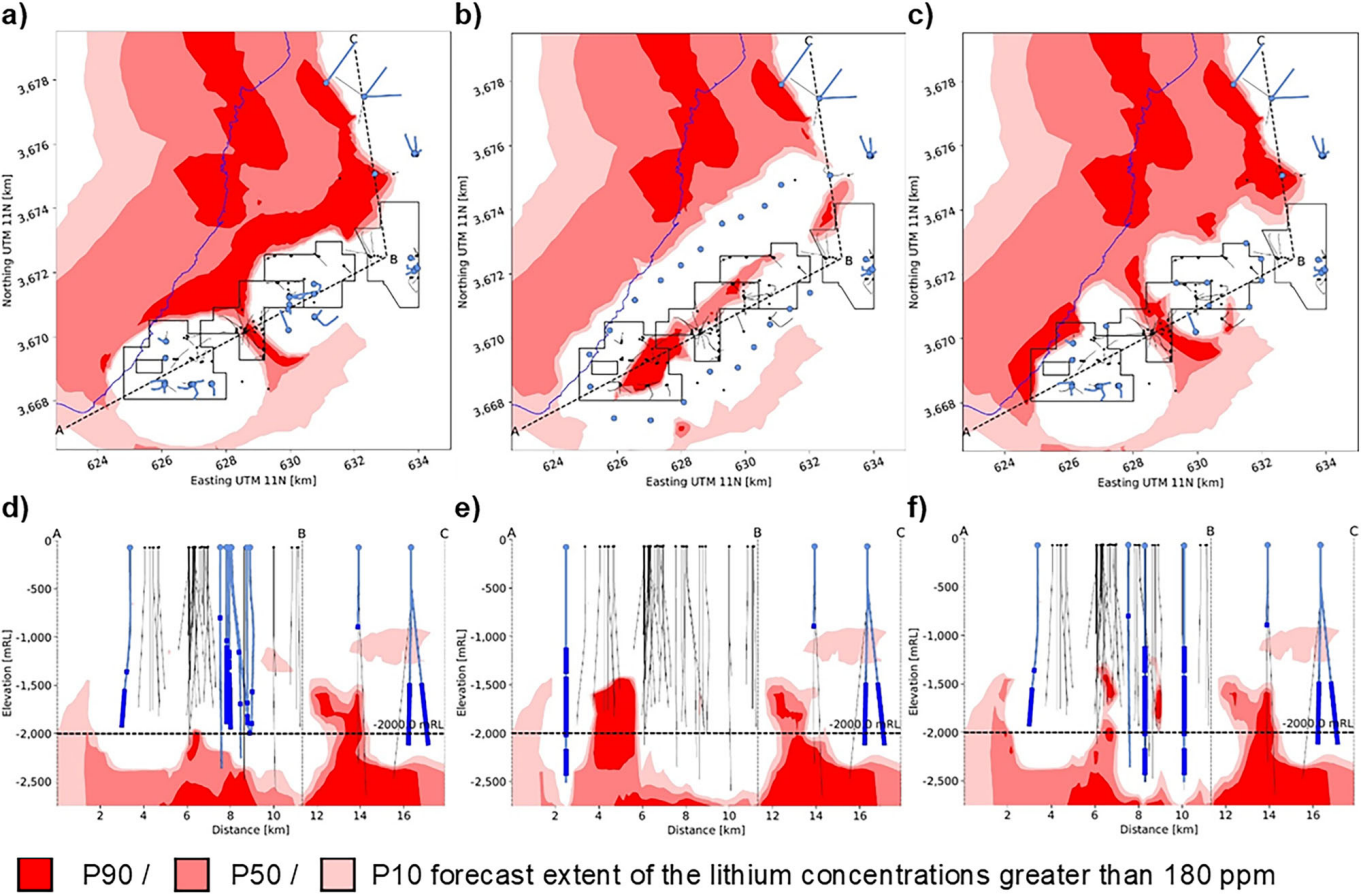
Map of well fields at Salton Sea and probabilistic distributions of the lithium concentration above 180 ppm in 2035 for the three reinjection scenarios. **a**, **d** Business-as-usual scenario. **b**, **e**Optimized outfield reinjection scenario. **c**, **f** Infield reinjection with local optimization scenario. Note: The maps and graphs show the probabilistic distributions of the lithium concentration above 180 ppm at −2000 mRL. Production wells and region boundaries are shown in black, reinjection wells in light blue and the Salton Sea coast in blue. P10, P50, and P90 are statistical percentiles shown as light, medium, and dark red.

Our process-based TEA model for large-scale lithium production from geothermal brines integrates multiple data sources (see Methods). The analysis begins with primary measurements and simulation data on future lithium concentrations across seven well field properties in the Salton Sea region, Fig. 1^21^. For each property, evaluate three confidence intervals, 90%, 50%, and 10% (P90/P50/P10), with P90 lithium concentrations ranging from 136.45 parts per million (ppm) at the Vulcan BN Geothermal Power Co. site to 198.21 ppm at CTR’s Hell’s Kitchen property.

We then assess lithium production costs under three operational scenarios for brine extraction and reinjection, namely business-as-usual, optimized outfield reinjection, and infield reinjection with local optimization. All scenarios assume a 30-year life-of-mine (LOM). This framework enables a consistent comparison of resource variability, operational strategies, and their combined impact on production costs.

The three 30-year operational scenarios, beginning in 2025, were evaluated using current production and reinjection data and assuming the future implementation of direct lithium extraction (DLE) at 95% recovery efficiency. Production strategies were held constant, with each well maintaining their current production rates and no new wells added, an approach that reflects present operations but may not capture future field development. Across all scenarios, total reinjection volumes were fixed at current levels, while the reinjection strategies varied. The first scenario, the business-as-usual scenario (006), extends the existing production and reinjection schedule. The second, optimized outfield reinjection (007), reallocates reinjection at the periphery of the lithium-rich geothermal plume to enhance lithium sweep while maintaining reservoir pressure. The third scenario, infield reinjection with local optimization scenario (008), shifts reinjection within three central production zones toward their boundaries to increase chemical breakthrough times and maximize local lithium recovery.

For each of the seven properties considered, the total lithium content dilution over time for the P90 brine production and reinjection scenario (in ppm), as well as individual facility’s brine throughputs, are listed Table 1. Supplementary Fig. 1 shows the development of lithium content over time for each scenario and property in kt LCE and brine throughput that remains constant over time (in Mt a^−1^). More detailed information on operational scenarios is provided in Supplementary Tables 1–3.

**Table 1:** Salton Sea region’s property data on brine throughput and lithium concentration under operational pumping and reinjection scenarios for large-scale lithium production facilities

No.		(1)	(2)	(3)	(4)	(5)	(6)	(7)
Property name		John L. Featherstone-Hudson Ran.	JM Leathers LP	Del Ranch LP	JJ Elmore LP	Vulcan BN Geothermal Power Co.	Region 1 Salton Sea Power Gener.	CTR-Hell’s Kitchen
Life-of-mine (year)		30	30	30	30	30	30	30
Brine throughput (kt a^−1^)		13,824	13,536	18,720	13,824	3600	38,160	15,264
Li ppm (scenario 006)	2025	174.07	179.63	195.32	167.6	136.45	155.28	198.21
	2040	79.38	64.88	60.33	98.1	65.66	60.73	65.16
	2054	53.77	36.12	40.57	73.67	47.84	43.53	53.26
Li ppm (scenario 007)	2025	174.07	179.63	195.32	167.6	136.45	155.28	198.21
	2040	70.77	79.29	92.07	93.27	84.47	79.59	67.68
	2054	43.66	35.1	48.9	59.54	58.5	54.03	51.84
Li ppm (scenario 008)	2025	174.07	179.63	195.32	167.6	136.45	155.28	198.21
	2040	84.54	47.84	98.31	76.97	81.91	60.93	66.48
	2054	50.77	25.69	66.6	58.02	62.51	44.32	55.11

Detailed information on each property and scenario, including probability considerations (P90/P50/P10), is shown in Supplementary Fig. 1 and Supplementary Tables 1–3. JM is not an abbreviation and is a part of the name of the geothermal well. LP refers to limited partnership, BN refers to the Brawley and Niland geothermal resource areas in Imperial County, California, where the plant operates, and CTR refers to Controlled Thermal Resources.

**Fig. 2:**
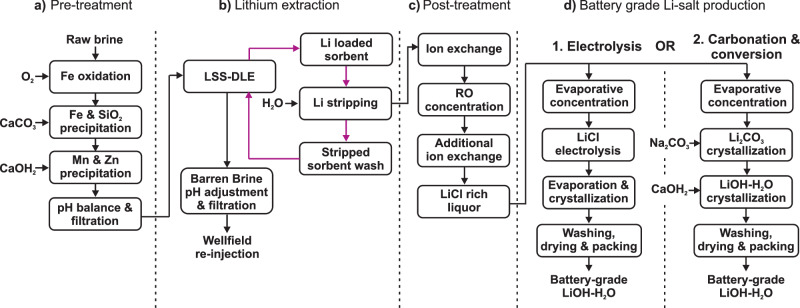
Modeled process flow diagrams (PFDs) for two process routes of large-scale lithium hydroxide monohydrate (LHM) production from geothermal brines at Salton Sea. **a** Pre-treatment of raw geothermal brine to remove impurities of iron (Fe), silicates (SiO_2_), manganese (Mn), and zinc (Zn). **b** Lithium extraction by lithium-selective sorption (LSS) as applied direct lithium extraction (DLE) technology, followed by lithium stripping from sorbent with water. **c** Post-treatment (or purification) of LiCl solution by ion-exchange (IX) steps to remove remaining impurities and reverse osmosis (RO) to further concentrate the LiCl solution. **d** Two optional pathways of battery-grade Li-salt production (lithium hydroxide monohydrate (LHM), LiOH•H_2_O): (1) Electrolysis of LiCl solution, followed by crystallization, washing and drying of LHM product, or (2) carbonation of LiCl with sodium carbonate (Na_2_CO_3_, “soda ash”) and precipitation of lithium carbonate (Li_2_CO_3_), followed by conversion to LHM with calcium hydroxide Ca(OH)_2_, “slacked lime”).

Next, for operating a large-scale lithium production facility on these properties, we modeled two different lithium salt production processes, both producing lithium hydroxide monohydrate LiOH•H_2_O (LHM) via (1) a carbonation and conversion and (2) an electrolysis pathway. After pumping underground brine, both production processes follow a chemical pre-treatment to precipitate major impurities, such as zinc and manganese, a lithium extraction step by lithium-selective sorption (a DLE technology), and further purification steps by ion exchange before final LHM production, as shown in the process flow diagrams (PFDs) in Fig. 2. The modeling of PFDs and associated production process costs are based on primary company data for LHM production from brines, and on patented processes for the pre-treatment of geothermal brines at Salton Sea (see “Methods”), which is highly sensitive to individual brine characteristics, such as metal contents, temperature, and pH value.

**Fig. 3:**
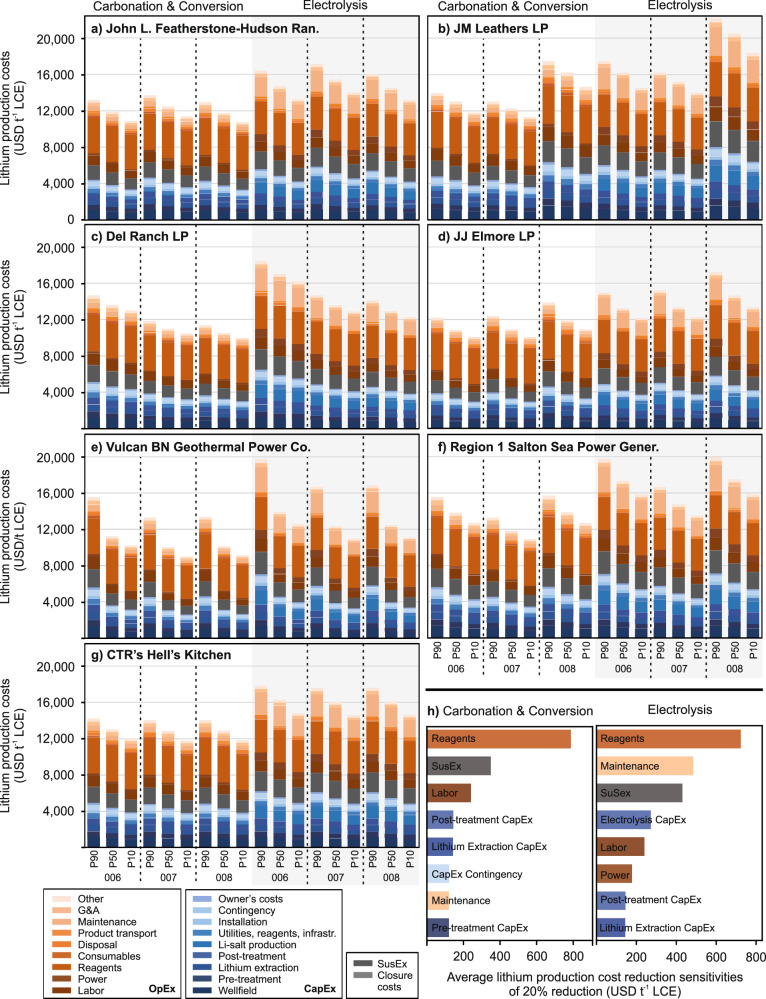
Baseline large-scale lithium production costs (in USD t^−1^ LCE) for each property, operating scenario (006/007/008), and lithium salt production process (carbonation and conversion/electrolysis) for P90/P50/P10. **a** John L. Featherstone-Hudson Ranch property**. b** JM Leathers LP property. **c** Del Ranch LP property. **d** JJ Elmore LP property. **e** Vulcan BN Geothermal Power Co. property. **f** Region 1 Salton Sea Power Gener. property. **g** CTR’s Hell’s Kitchen property. **h** Top eight sensitivities for carbonation & conversion and electrolysis process route, expressed as impacts of 20% reductions of individual cost driver. Note:“006” represents the business-as-usual scenario,”007” is the optimized outfield reinjection scenario, and “008” shows the infield reinjection with local optimization scenario. Note: Baseline lithium production costs (USD t^−1^ LCE) for each scenario are presented in Supplementary Table 4. Note: JM is not an abbreviation and simply a part of the name of the geothermal well. LP refers to limited partnership, BN refers to the Brawley and Niland geothermal resource areas in Imperial County, California, where the plant operates, and CTR refers to Controlled Thermal Resources.

Total costs of large-scale LHM production (in USD t^−1^ LCE) on each property are presented in Fig. 3, with total costs structured by the main cost categories of CapEx, OpEx, SusEx, and mine closure costs. In addition, to identify major cost drivers that provide strategic pathways for process costs optimization, Fig. 3h shows sensitivities of granular cost categories of both LHM production processes (average sensitivities across all properties under 006/P90 scenarios).

The results show that across all scenarios, electrolysis is constantly more expensive than the carbonation and conversion production pathways, mainly driven by substantial increases in costs of maintenance, power, and CapEx for the final LHM production step (i.e., electrolyzers). In general, as for carbonation and conversion processes, P90 production costs vary from around 11,355 USD t^−1^ LCE (008) on the Del Ranch LP property to 17,466 USD t^−1^ LCE (008) on the JM Leathers LP property (P10: 9000 USD t^−1^ LCE for Vulcan BN Geothermal Power Co./007 to 14,610 USD t^−1^ LCE for JM Leathers LP/008). For electrolysis processes, P90 costs range from 14,062 to 22,153 USD t^−1^ LCE (P10: 10,947 to 18,354 USD t^−1^ LCE), respectively to the properties and operational scenarios.

Setting these costs into context reveals significant gaps. Recent industry cost-curve analyses indicate that, in the current market, high-grade continental brines represent the lowest-cost production pathway (~4000–6000 USD t^−1^ LCE), while hard-rock spodumene operations span a wide range (~5000–15,000 USD t^−1^ LCE) depending on deposit grade, scale, and degree of vertical integration, with top-tier integrated projects (e.g., Greenbushes) remaining competitive and average producers operating at the higher end^13,22,23^. Clay-hosted resources, exemplified by the Thacker Pass project, have matured to cash costs/all-In Sustaining Costs (C1/AISC) estimates of ~6200–7500 USD t^−1^ LCE^24^. Against these benchmarks, Salton Sea geothermal brines, given their lower lithium concentrations (~150–200 mg L^−1^) compared with South American Salars (~1500–2500 mg L^−1^), face a substantial cost gap and require targeted optimization to become long-term cost-competitive. Moreover, although production costs decrease with higher lithium content from P90 to P10 in a given operational scenario, they hardly fall below current lithium prices of around 10,000 USD t^−1^ LCE^14^, challenging the overall economic viability of lithium production facilities at Salton Sea under volatile market conditions. Therefore, based on the identified major cost drivers, we investigated potential optimization pathways towards economic viability with respect to dynamic lithium price environments.

### Scenario analysis for strategic pathways to economic viability

The three main costs drivers for total lithium production costs identified in the sensitivity analysis (see Fig. 3h) are reagents costs (mainly driven by costs for calcium hydroxide (Ca(OH)_2_) and calcium carbonate (CaCO_3_) in the pre-treatment processes to precipitate the impurity metals zinc and manganese), CapEx for processing plants, and costs of labor and facility maintenance. We note that some of these costs may be covered by existing geothermal power operations, which incorporate some degree of brine pretreatment prior to reinjection. Moreover, the developed portion of the geothermal field already has the wellfield, pipelines, and pumping facilities, so those costs would not be borne by adding lithium recovery to these portions of the field. Based on these major cost drivers, we analyzed the impact of three scenarios on the large-scale facilities’ long-term economic viability, i.e., net present value (NPV). These three scenarios are: By-product valorization, financial subsidies for CapEx, and process optimization and automation reducing labor and maintenance costs. More specifically, to account for volatile market environments, we determined dynamic ‘break-even’ lithium prices (lithium price for NPV = 0) in each scenario that represent minimal market price threshold conditions, under which the facilities can be operated profitably in the long term.

**Fig. 4:**
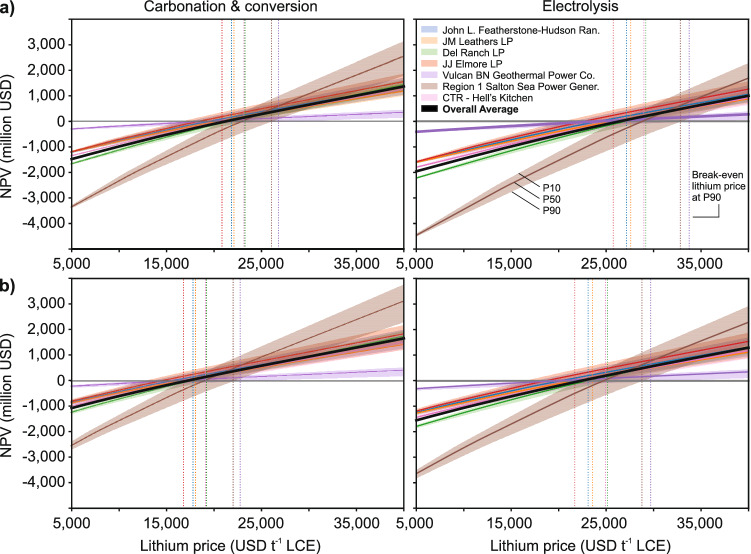
Viability or net present value (NPV) (in million USD) of large-scale lithium production at Salton Sea under lithium prices from 5000 to 40,000 USD t^−1^ LCE for the business-as-usual (006) scenario. **a** NPV without by-product revenue. **b** NPVs including by-product revenues of precipitated Zn(OH)_2_, Mn(OH)_2_, and CaCl_2_ in pre-treatment process. Note: Dotted lined represent break-even lithium price (NPV = 0) for P90.

Figure 4 compares the resulting basic NPV of lithium production facilities on each property (P90/business-as-usual 006) for long-term lithium prices between 5,000 and 40,000 USD t^−1^ LCE for carbonation and conversion and electrolysis process facilities, assuming no by-product sales, Fig. 4a, and by-product sales of precipitated zinc hydroxide, manganese hydroxide, and calcium chloride (Zn(OH)_2_, Mn(OH)_2_, and CaCl_2_) in brine pre-treatment, Fig. 4b. Detailed NPV values for specific lithium prices and ‘break-even’ lithium prices for all scenarios are listed Supplementary Tables 5 and 6.

In general, the Region 1 Salton Sea Power Generation property facility shows both lowest and highest potential NPV across all properties and scenarios caused by its high lithium production rate potential compared to the other properties (over 30,000 t LCE in year 1, see Supplementary Fig. 1), and thus, highest revenue/loss potentials under extreme lithium market prices. However, the minimum break-even lithium prices under most conservative cost conditions (P90/006) without by-product sales are 20,876 USD t^−1^ LCE for a carbonation and conversion process, and 25,478 USD t^−1^ LCE for LHM production by electrolysis on the JJ Elmore LP property, while maximum break-even prices are 26,806 and 33,747 USD t^−1^ LCE on the Vulcan BN Geothermal Power Co. property, respectively, Fig. 4a. Considering by-product sales of precipitated Zn(OH)_2_, Mn(OH)_2_, and CaCl_2_ in pre-treatment, thereby leveraging high reagent consumption of CaCO_3_ and Ca(OH)_2_, minimum break-even prices are reduced to 16,825 (−20%) and 21,696 USD t^−1^ LCE (−16%), for carbonation and conversion and electrolysis facilities on the JJ Elmore LP property, respectively.

**Fig. 5:**
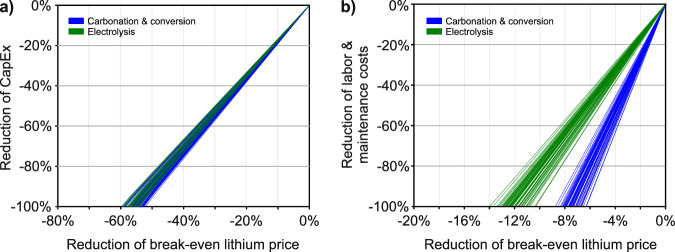
Impact of capital expenditure (CapEx) and labor/maintenance cost reduction on break-even lithium prices (NPV = 0) for long-term large-scale lithium production viability. **a** Change in CapEx (%) vs. change in break-even lithium price (%) for all scenarios (006/007/008; P10/P50/P90). **b** Change in labor/maintenance costs (%) vs. change in break-even lithium price (%) for all scenarios (006/007/008; P10/P50/P90).

For assessing the viability impacts of CapEx subsidies and labor/maintenance improvements, we analyzed reducing both cost categories as function of the basic costs (in %), and their effects on reducing basic break-even lithium price (in %) in each operational scenario on all properties (Fig. 5). Figure 5a shows that, for example, a reduction of CapEx by 50% potentially reduces the break-even lithium price by around 30%, varying only marginally between both production processes and across all scenarios. For example, a CapEx subsidy of around 410 million USD to a facility installed on the JJ Elmore LP property applying a carbonation and conversion process (−50% from basic ~820 million USD; 006/P90), could reduce the long-term, minimum break-even lithium price, above which this facility is viable, from 20,876 (or 16,825 incl. by-product sales) to around 14,610 (or 11,780) USD t^−1^ LCE (−30%).

Compared to CapEx subsidies, however, optimizing labor and maintenance costs shows significantly lower cost reduction potentials, leading to decreased break-even lithium prices of around 4% for carbonation and conversion facilities, and 6% for electrolysis facilities for a 50% improvement in costs, on average across all scenarios (Fig. 5b).

## Discussion

This study provides a comprehensive techno-economic assessment of large-scale lithium production from geothermal brines in California’s Salton Sea region, addressing a central question in the debate over alternative lithium supply pathways. By integrating primary lithium concentration measurements, reservoir simulations, process patents, and company cost data, we develop a process-based model that reflects realistic operating and market conditions.

Across seven properties, three operational scenarios, and two downstream processing routes, our results indicate that most modeled production costs exceed prevailing lithium prices (10,000 USD t^−1^ LCE). Even under favorable resource assumptions (P90), the lowest cost estimate is 11,355 USD t^−1^ LCE. Electrolysis-based pathways remain consistently more expensive than carbonation-and-conversion due to their higher capital and operational demands. These findings point to three primary cost drivers: reagent-intensive impurity removal, substantial capital investment for processing infrastructure, and recurring labor and maintenance requirements.

We identify several pathways that materially improve economic performance. Revenues from by-product recovery (Zn(OH)_2_, Mn(OH)_2_, CaCl_2_) can reduce break-even prices by ~12–20%, even after conservative deductions for processing and transport losses. This aligns with established industry practice, where by-products often strengthen project economics in multi-metal systems^25,26^. Public support mechanisms, such as concessional financing or capital grants, offer another powerful lever^27^. A representative 450 million USD subsidy could lower break-even prices by ~23–30%, consistent with recent U.S. federal investments in domestic lithium projects. Operational efficiencies, including automation and predictive maintenance, provide more modest gains but remain meaningful, particularly for electrolysis facilities. These results suggest that while geothermal brines hold promise as a low-footprint, co-located source of thermal power and lithium, their economic viability hinges on strategic cost reductions and supportive policy environments. Importantly, our break-even analysis provides threshold price conditions under which geothermal projects become commercially feasible, offering actionable guidance for developers and policymakers.

The study has limitations. Certain cost inputs rely on analogs from other brine systems or geothermal regions, introducing uncertainty in transferability. For example, individual impurity characteristics of brines may impact capital expenditure for DLE plants. Moreover, lithium dilution behavior remains a key sensitivity, and future work incorporating more detailed reservoir models will improve long-term cost estimates^28^. Nonetheless, the framework presented here, combining resource characterization, operational scenarios, and dynamic cost modeling, provides a scalable approach for evaluating emerging critical material production pathways.

**Fig. 6:**
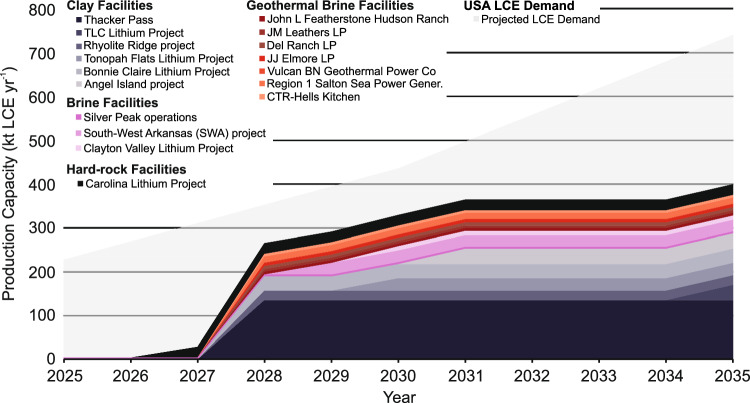
Future lithium supply and demand comparison in the United States considering geothermal brine facilities and currently announced, developed, and operating lithium production facilities based on hard-rock ore, brine, and clay resources. Note: See Supplemental Note 2 for calculation methodology for domestic lithium demand projections. Values for lithium production from all non-geothermal resources are taken from ref. ^8^.

When contextualized against projected U.S. lithium demand^8^, our results indicate that even full development of all seven Salton Sea properties, in conjunction with prior findings on future lithium supply in the United States, would meet only a fraction of future domestic requirements (Fig. 6). If geothermal production at the Salton Sea Geothermal Field were to be expanded, increased lithium production could result, leading to a narrowing of this gap. The current gap, however, highlights the need for continued technological innovation, diversified resource development, and integrated analyses that link production costs with national supply–demand trajectories and supply chain risk.

Looking ahead, future research could expand feasibility analysis by integrating evolving, yet lower technology-readiness-level DLE methods, such as solvent extraction and electrochemical DLE, and should couple techno-economic modeling with broader market dynamics and cross-material risk assessments. Such integrated approaches will be essential for jurisdictions, including the United States, Europe, India, and Australia, that remain vulnerable to concentrated critical material supply chains. By bringing together resource characterization, long-term operational modeling, process-level cost analysis, and market risk evaluation, the framework developed here can be extended to assess other emerging pathways for critical material production.

## Methods

### Facility cost analysis approach

In this study, we combine multiple primary data sets for calculating large-scale lithium production facility costs based on geothermal brine resources using the case of the Salton Sea region in California. Thereby, we investigate effects of various operational brine pumping and reinjection scenarios on a total of seven different properties in the region. Our cost analysis is based on (1) primary measurement and simulation data for property-specific lithium content in pumped brine over time under operational scenarios, (2) process patents for the pre-treatment of pumped brine, and (3) primary cost data of companies applying DLE and lithium hydroxide monohydrate (LHM) production processes to lithium-bearing brines in North America and Europe.

### Operational scenarios and brine characterization

As a key input parameter, the cost model in this study applies brine lithium concentration developments over time for seven properties in the Salton Sea region (see Fig. 1 and Table 1)^21^. Data on lithium concentration in each property are obtained from prior studies that measured basic lithium contents (in ppm) and simulated three operational scenarios over time (business-as-usual “006”, global optimization “007”, and local optimization “008”), characterized by different operational parameters that determine fresh brine pumping rate and spent brine reinjection rate after processing, which affects lithium content in pumped brine across the simulated time frame of 30 years (for more details and operational scenario data, see Supplementary Tables 1–3). Moreover, to account for uncertainties, the lithium content measurements and in-time simulations provide lithium content confidence intervals of 10%, 50%, and 90%, which we integrate in the cost analysis in this study as P10/P50/P90 scenarios. Accordingly, P90 shows the lowest and P10 the highest overall lithium content within a respective operational scenario and property setting.

More precisely, the operational model for brine production and reinjection is based on a high-resolution, three-dimensional reservoir model of the Salton Sea Geothermal Field using the Waiwera geothermal simulator. The conceptual model integrated geological, structural, and geophysical data, including fault systems and stratigraphy, to capture the field’s heterogeneity. A dual-porosity approach was applied to represent fracture-dominated flow, with lithium treated as a conservative tracer. Calibration combined natural-state and production-history simulations using historical data from over 150 wells, including temperature, chemistry, and reinjection records. Boundary conditions accounted for deep upflow zones and hydrostatic conditions beneath the Salton Sea. To address uncertainty, a probabilistic ensemble of 1000 models was generated and filtered using Approximate Bayesian Computation, resulting in 100 calibrated models for forecasting. These models were used to evaluate lithium recovery and geothermal performance under three operational scenarios, incorporating variability in permeability, porosity, and fluid connectivity.

### Patent data for brine pre-treatment process

After pumping brines to facilities’ surface plants, pre-treatment is the first critical step in the process of producing LHM from geothermal brines. Prior to lithium extraction by DLE, this step removes large amounts of impurity materials in the brine, such as iron, silicates, zinc, and manganese, to enable selective extraction and increase efficiency of following purification and final LHM production process steps. Pre-treatment processes applied to brines are highly sensitive to individual brine characteristics, such as quality and quantity of impurities, temperature, or pH of brines that vary significantly between regional geothermal brine resources. In this study, to model pre-treatment process step requirements and associated costs (e.g., for chemical reagents used to remove impurities or regulate pH), we apply process patents describing pre-treatment of geothermal brine in the Salton Sea region by precipitating iron silicates, zinc hydroxide (Zn(OH)_2_), and manganese hydroxide (Mn(OH)_2_)^29^. By stoichiometric balances in the chemical reactions and impurity quantity data provided in the brine characterization model, we further derived required reagent consumption in the pre-treatment processes of large-scale facilities for each property and operational scenarios, respectively, to eventually determine overall costs of pre-treatment processes (for more details on the patent process, stoichiometry of chemical reactions, total reagent consumption in pre-treatment processes, and reagent cost assumptions see Supplementary Note 1).

### Process modeling and primary cost data

For modeling lithium extraction by DLE after pre-treatment and final LHM production, we draw on lithium production processes based on brines applied by companies in North America and Europe (see Supplementary Table 7 for an overview of considered companies and primary data sources). Based on these companies’ applied processes, we modeled two PFDs for LHM production (see Fig. 2). In both processes, after pre-treatment, lithium is extracted by a DLE technology based on lithium-selective sorption by an alumina-based sorbent, which is stripped with water consequently, followed by further purification steps of selective ion-exchange (IX) to remove remaining, small-scale impurities from the lithium chloride (LiCl) solution and a reverse osmosis step to concentrate the solution. The two processes modeled in this study differ in the final step of producing LHM. First, we model a reagent-intensive carbonation and conversion production step, where lithium is first precipitated as lithium carbonate (Li_2_CO_3_) using sodium carbonate (Na_2_CO_3_, or “soda ash”) and then converted to LHM (LiOH x H_2_O) with calcium hydroxide (Ca(OH)_2_, or “slacked lime”). In the second, alternative process, the purified LiCl solution undergoes an energy-intensive electrolysis process to produce a high-purity LiOH-solution that must be evaporated and dried, ultimately.

For the process-based, large-scale facility cost model in this study, we consider total project costs across the entire life-of-mine (LOM) of 30 years (based on brine characterization model data scope). For analyzing total LOM project costs, we apply a total project cost categorization methodology used in prior studies^8,13^, including total projects’ initial capital expenditures (CapEx; ${{CapEx}}_{{total}}^{i}$), LOM operating expenditures (OpEx; ${{OpEx}}_{{total}}^{i}$), LOM sustaining capital expenditures (SusEx; ${{SusEx}}_{{total}}^{i}$), and closure costs of mines/facilities (${{Costs}}_{{closure}}^{i}$) after LOM. Aggregating all costs results in the total costs of lithium production for a respective project i, characterized by considered Salton Sea region property, operational scenario, lithium content confidence interval, and applied production process, as shown in Eq. (1):

1 $${{Costs}}_{{total}}^{i}\,\left[{USD}\right]=	\, {{CapEx}}_{{total}}^{i}\left[{USD}\right]+{{OpEx}}_{{total}}^{i}\,\left[{USD}\right] \\ 	+{{SusEx}}_{{total}}^{i}\left[{USD}\right]+{{Costs}}_{{closure}}^{i}[{USD}]$$

In our model, we further break down each cost category of CapEx, OpEx, and SusEx into individual cost sub-categories. CapEx, for example, includes expenditures for wellfield drilling and pumping equipment, pre-treatment plants, DLE and purification plants, and final processing facilities, as well as for reagent tanks, utility and infrastructure, and indirect costs (e.g., owner’s costs and contingencies). OpEx includes costs for reagents, labor, power, maintenance, consumables, waste disposal, product transport, and general and administrative tasks (G&A). Finally, SusEx include, for example, replacement of membranes, filters, IX resins, and electrolysis units.

Large-scale production cost data for these sub-categories are based on primary cost data of the North American and European companies analyzed for modelling in this study. To derive values within each cost category for the modeled large-scale lithium production facilities based on the geothermal brine at Salton Sea, we quantified cost category averages based on the companies’ primary cost data set. More specifically, we derive cost averages based on two distinguished key scaling factors. The first scale factor, total LOM lithium production (in kt LCE per year), mainly determines most OpEx categories, such as reagents (including pre-treatment reagents), consumables, products transport, and waste disposal. The second scale factor, total LOM geothermal brine volume or throughput (in Mt brine per year), affects CapEx, SusEx, and individual OpEx categories. For example, sizes of processing plants correlate with the volume of brine throughput, whereas individual OpEx (e.g., labor and power) scale with facility size to enable operations.

In sum, in the large-scale, lithium production process-based cost model in this study, we first derive average cost category scale factors based on primary company cost data (e.g., average CapEx for DLE plants in USD Mt^−1^ brine throughput; or average CaCO_3_ pre-treatment reagent costs in USD t^−1^ LCE). Second, applying these average scale factors, we model geothermal facility costs based on respective total LCE production and brine throughput for each property, lithium content certainty, and operational scenarios (see Supplementary Fig. 1 and Supplementary Tables 1–3). More details on cost values within cost categories for each company considered in this study, as well as on derived average cost scale factors used to calculate geothermal facility costs are shown in the Supplementary Tables 7 and 8.

### Economic viability of large-scale production

To assess economic viability, we calculate the NPVs of each of the large-scale lithium production facilities modeled in this study. The NPV accounts for the total projects economic viability by summarizing discounting total annual cash flows throughout the LOM. The NPV calculation is based on modeled total project costs (up-front CapEx, annual OpEx, required SusEx across LOM, closure costs after LOM), annual lithium production rates, lithium market prices, and other financial parameter, such U.S. federal tax rates, California state tax rates, property tax rates, annual discount rates, and California State lithium extraction tax (for more details on NPV calculation parameter settings and by-product revenue estimates, see Supplementary Tables 9 and 10).

In this study, rather than calculating NPVs for fixed lithium prices, we mainly investigate individual modeled lithium production project’s NPVs as a function of dynamic lithium prices. Throughout the scenario analysis of project’s economic viability, we determine lithium prices where NPV = 0 (“break-even” lithium prices) that present minimum viable lithium market conditions to enable profitable facility operations in the long term.

## Supplementary information

Supplementary Information

Transparent Peer Review file

## Data Availability

The authors declare that the main data supporting the findings of this study are available within the article and its Supplementary Information files.
