# Supplementary material for: Pathways to cost competitive and viable lithium production from Salton Sea geothermal brines
Source: Nat Commun. 2026 Jul 9;17:8448. doi: 10.1038/s41467-026-75389-8 (PMC13478165; doi:10.1038/s41467-026-75389-8)
Supplement: Supplementary file 1 [file 41467_2026_75389_MOESM1_ESM.pdf]

Supplementary Information to the article:

Pathways to Cost Competitive and Viable Lithium Production from Salton Sea Geothermal Brines

Jannis Wesselkaemper[1*], Theo Renaud[2], Naod Araya[2], Ken Dekkers[2], Joris Popineau[2], Jeremy Riffault[2], John O'Sullivan[2], Andrew Z. Haddad[3*]

Correspondence to: azhaddad@lbl.gov; janniswesselkaemper@gmail.com

[1]*Energy Analysis and Environmental Impacts Division, Energy Technologies Area, Lawrence Berkeley National Laboratory, Berkeley, CA, USA*

[2]*Geothermal Institute, University of Auckland, 70 Symonds Street, Grafton, Auckland, 1010, New Zealand*

[3]*Energy Storage and Distributed Resources Division, Energy Technologies Area, Lawrence Berkeley National Laboratory, Berkeley, CA, USA*

Table of Contents

Supplementary Figures

Supplementary Figure 1: Lithium content in brine (in kt LCE in pumped brine) and total brine throughput (in Mt per year) in each scenario (P90/P50/P10) for large-scale facilities on Salton Sea properties for total life-of-mine (LOM) from 2025 to 205……………………………………………..**2**

Supplementary Tables

Supplementary Table 1: Salton Sea region's property data on brine throughput and lithium concentration (in t LCE/a) under operational pumping and reinjection scenarios for large-scale lithium production facilities for P90…………………………………………………………………..**2**

Supplementary Table 2: Salton Sea region's property data on brine throughput and lithium concentration (in t LCE/a) under operational pumping and reinjection scenarios for large-scale lithium production facilities for P50…………………………………………………………………..**3**

Supplementary Table 3: Salton Sea region's property data on brine throughput and lithium concentration (in t LCE/a) under operational pumping and reinjection scenarios for large-scale lithium production facilities for P10…………………………………………………………………..**3**

Supplementary Table 4: Baseline lithium production costs (USD/t LCE) for each scenario………**4**

Supplementary Table 5: Net present values (NPVs) (in million USD) for each scenario under lithium prices of 10,000, 20,000 and 30,000 USD/t LCE……………………………………………..**5**

Supplementary Table 6: Break-even (NPV=0) lithium prices (USD/t LCE) for each scenario…..…**7**

Supplementary Table 7: Details on considered lithium production projects from brines in USA, Canada, and Germany for large-scale process and costs modelling in this study………………..….**8**

Supplementary Table 8: Average scale factors for modelling cost categories of large-scale lithium extraction from geothermal brines in the Salton Sea area in this study derived from average project cost categories of projects presented in Supplementary Table 7 above…………………………....**9**

Supplementary Table 9: Parameter applied for NPV calculations in this study for the Salton Sea region in California……………………………………………………………………………………**9**

Supplementary Table 10: Parameter applied for by-product revenue calculation……………..….**10**

Supplementary Notes:

Supplementary Note 1: Pre-treatment reagent cost calculations methodology: Chemical reactions for precipitating impurities in Salton Sea geothermal brine (manganese, zinc, iron, silicate) during pre-treatment processes, reagent consumption, calculation parameters, and reagent purchase prices……………………………………………………………………………………………**10**

Supplementary Note 2: Calculation logic and methodology for projected domestic lithium demand……………………………………………………………………………………………**11**

Supplementary References…………………………………………………………………………...**12**

Supplementary Figure 1: Lithium content in brine (in kt LCE in pumped brine) and total brine throughput (in Mt per year) in each scenario (P90/P50/P10) for large-scale facilities on Salton Sea properties for total life-of-mine (LOM) from 2025 to 2054. Note: '006' represent the business-as-usual scenario, '007' is the optimized outfield reinjection scenario, and '008' shows the infield reinjection with local optimization scenario. Lithium content in brine is diluted over time due to production and reinjection of brine after extraction, whereas brine throughput remains constant over time in the modelled operational scenarios. Note: JM is not an abbreviation and simply a part of the name of the geothermal well. LP refers to limited partnership, BN refers to the Brawley and Niland geothermal resource areas in Imperial County, California, where the plant operates, and CTR refers to Controlled Thermal Resources.

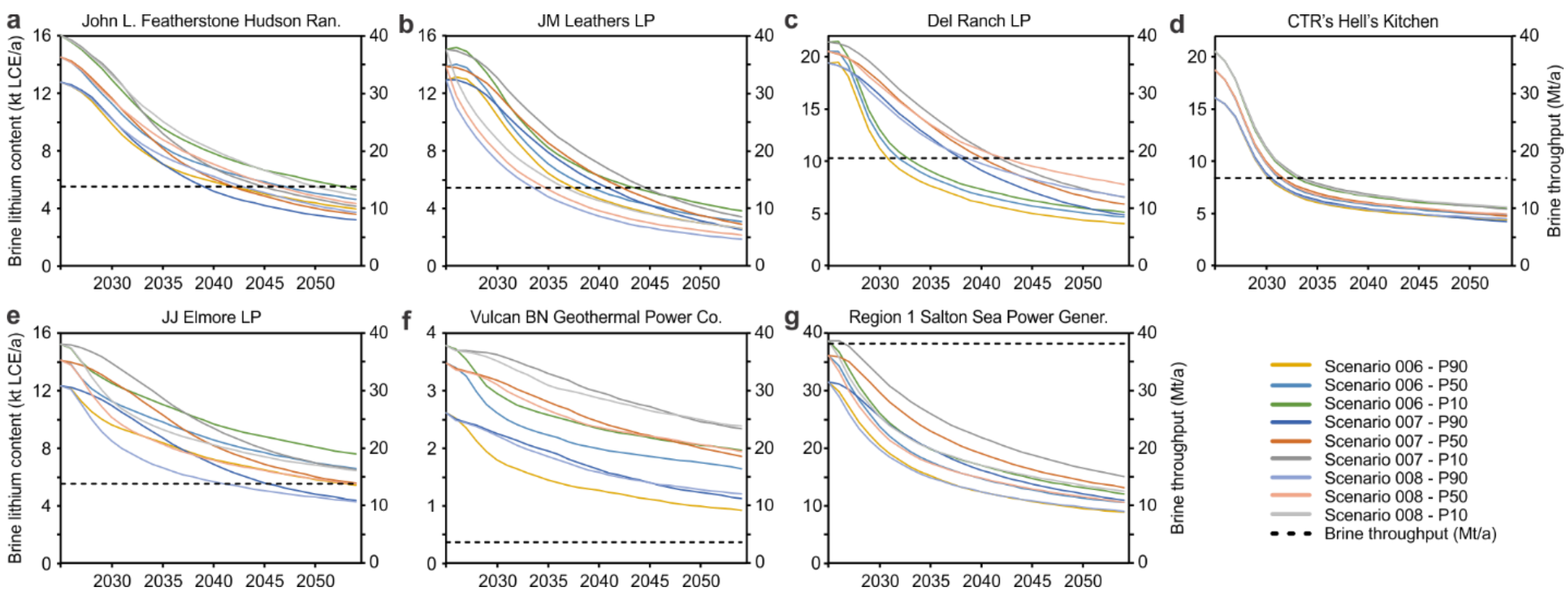

Supplementary Table 1: Salton Sea region's property data on brine throughput and lithium concentration (in t LCE/a) under operational pumping and reinjection scenarios for large-scale lithium production facilities for P90. Note: '006' represent the business-as-usual scenario, '007' is the optimized outfield reinjection scenario, and '008' shows the infield reinjection with local optimization scenario. Note: JM is not an abbreviation and simply a part of the name of the geothermal well. LP refers to limited partnership, BN refers to the Brawley and Niland geothermal resource areas in Imperial County, California, where the plant operates, and CTR refers to Controlled Thermal Resources.

No.		1)	2)	3)	4)	5)	6)	7)
Property name		John L. Featherstone-Hudson Ran.	JM Leathers LP	Del Ranch LP	JJ Elmore LP	Vulcan BN Geothermal Power Co.	Region 1 Salton Sea Power Gener.	CTR-Hell's Kitchen
Life-of-mine (year)		30	30	30	30	30	30	30
Brine throughput (kt/a)		13,824	13,536	18,720	13,824	3,600	38,160	15,264
t LCE/a	2025	12,809	12,943	19,463	12,333	2,615	31,541	16,105
(scenario	2040	5,841	4,675	6,012	7,219	1,258	12,336	5,294
006)	2054	3,957	2,603	4,043	5,421	917	8,842	4,327
t LCE/a	2025	12,809	12,943	19,463	12,333	2,615	31,541	16,105
(scenario	2040	5,208	5,713	9,174	6,863	1,619	16,167	5,499
007)	2054	3,213	2,529	4,873	4,381	1,121	10,975	4,212
t LCE/a	2025	12,809	12,943	19,463	12,333	2,615	31,541	16,105
(scenario	2040	6,221	3,447	9,796	5,664	1,570	12,376	5,402
008)	2054	3,736	1,851	6,636	4,269	1,198	9,003	4,478

Supplementary Table 2: Salton Sea region's property data on brine throughput and lithium concentration (in t LCE/a) under operational pumping and reinjection scenarios for large-scale lithium production facilities for P50. Note: '006' represent the business-as-usual scenario, '007' is the optimized outfield reinjection scenario, and '008' shows the infield reinjection with local optimization scenario. Note: JM is not an abbreviation and simply a part of the name of the geothermal well. LP refers to limited partnership, BN refers to the Brawley and Niland geothermal resource areas in Imperial County, California, where the plant operates, and CTR refers to Controlled Thermal Resources.

**No.**		**1)**	**2)**	**3)**	**4)**	**5)**	**6)**	**7)**
Property name		John L. Featherstone-Hudson Ran.	JM Leathers LP	Del Ranch LP	JJ Elmore LP	Vulcan BN Geothermal Power Co.	Region 1 Salton Sea Power Gener.	CTR-Hell's Kitchen
Life-of-mine (year)		30	30	30	30	30	30	30
Brine throughput (kt/a)		13,824	13,536	18,720	13,824	3,600	38,160	15,264
t LCE/a	2025	14,510	13,896	20,534	14,074	3,475	36,065	18,729
(scenario	2040	6,801	5,217	6,774	8,576	1,984	14,637	5,868
006)	2054	4,624	3,079	4,669	6,578	1,635	10,504	4,797
t LCE/a	2025	14,510	13,896	20,534	14,074	3,475	36,065	18,729
(scenario	2040	6,047	6,234	10,346	8,186	2,452	19,019	6,062
007)	2054	3,575	2,885	5,896	5,577	1,847	13,156	4,756
t LCE/a	2025	14,510	13,896	20,534	14,074	3,475	36,065	18,729
(scenario	2040	7,064	3,833	11,092	7,135	2,355	14,747	5,987
008)	2054	4,336	2,154	7,810	5,504	1,936	10,621	4,934

Supplementary Table 3: Salton Sea region's property data on brine throughput and lithium concentration (in t LCE/a) under operational pumping and reinjection scenarios for large-scale lithium production facilities for P10. Note: '006' represent the business-as-usual scenario, '007' is the optimized outfield reinjection scenario, and '008' shows the infield reinjection with local optimization scenario.

**No.**		**1)**	**2)**	**3)**	**4)**	**5)**	**6)**	**7)**
Property name		John L. Featherstone-Hudson Ran.	JM Leathers LP	Del Ranch LP	JJ Elmore LP	Vulcan BN Geothermal Power Co.	Region 1 Salton Sea Power Gener.	CTR-Hell's Kitchen
Life-of-mine (year)		30	30	30	30	30	30	30
Brine throughput (kt/a)		13,824	13,536	18,720	13,824	3,600	38,160	15,264
t LCE/a	2025	16,045	15,094	21,475	15,225	3,774	38,671	20,512
(scenario	2040	7,845	6,269	7,328	9,680	2,336	16,967	6,671
006)	2054	5,334	3,819	5,151	7,573	1,965	12,072	5,493
t LCE/a	2025	16,045	15,094	21,475	15,225	3,774	38,671	20,512
(scenario	2040	6,792	7,126	11,243	9,353	2,977	21,842	6,881
007)	2054	4,158	3,383	6,528	6,496	2,330	15,078	5,418
t LCE/a	2025	16,045	15,094	21,475	15,225	3,774	38,671	20,512
(scenario	2040	8,086	4,554	12,191	8,222	2,869	17,002	6,757
008)	2054	4,932	2,606	8,727	6,436	2,379	12,521	5,603

Supplementary Table 4: Baseline lithium production costs (USD/t LCE) for each scenario.

Scenario			Baseline lithium production costs (USD/t LCE)	
**Well field property**	**Operation scenario**	**Confidence interval**	Carbonation & Conversion	Electrolysis
John L. Featherstone-Hudson Ran.	006	P90	13,193.6	16,412.1
John L. Featherstone-Hudson Ran.	006	P50	11,888.7	14,683.7
John L. Featherstone-Hudson Ran.	006	P10	10,804.5	13,247.7
John L. Featherstone-Hudson Ran.	007	P90	13,734.0	17,127.9
John L. Featherstone-Hudson Ran.	007	P50	12,452.9	15,431.1
John L. Featherstone-Hudson Ran.	007	P10	11,373.5	14,001.4
John L. Featherstone-Hudson Ran.	008	P90	12,937.2	16,072.5
John L. Featherstone-Hudson Ran.	008	P50	11,761.3	14,515.0
John L. Featherstone-Hudson Ran.	008	P10	10,747.1	13,171.7
JM Leathers LP	006	P90	13,945.8	17,469.8
JM Leathers LP	006	P50	13,004.4	16,217.5
JM Leathers LP	006	P10	11,727.9	14,519.4
JM Leathers LP	007	P90	13,010.3	16,225.4
JM Leathers LP	007	P50	12,238.2	15,198.2
JM Leathers LP	007	P10	11,327.5	13,986.7
JM Leathers LP	008	P90	17,466.4	22,153.3
JM Leathers LP	008	P50	16,210.8	20,482.9
JM Leathers LP	008	P10	14,610.3	18,353.8
Del Ranch LP	006	P90	14,645.1	18,453.1
Del Ranch LP	006	P50	13,587.4	17,041.2
Del Ranch LP	006	P10	12,941.8	16,179.3
Del Ranch LP	007	P90	11,804.7	14,661.3
Del Ranch LP	007	P50	10,979.6	13,559.9
Del Ranch LP	007	P10	10,447.3	12,849.2
Del Ranch LP	008	P90	11,355.4	14,061.6
Del Ranch LP	008	P50	10,516.9	12,942.2
Del Ranch LP	008	P10	9,978.8	12,223.9
JJ Elmore LP	006	P90	12,168.9	14,971.3
JJ Elmore LP	006	P50	10,842.1	13,226.4
JJ Elmore LP	006	P10	10,006.0	12,126.8
JJ Elmore LP	007	P90	12,347.9	15,206.7
JJ Elmore LP	007	P50	10,910.3	13,316.0
JJ Elmore LP	007	P10	10,077.3	12,220.6
JJ Elmore LP	008	P90	13,866.7	17,204.1
JJ Elmore LP	008	P50	11,900.1	14,617.8
JJ Elmore LP	008	P10	10,911.2	13,317.2
Vulcan BN Geothermal Power Co	006	P90	15,550.2	19,725.9
Vulcan BN Geothermal Power Co	006	P50	11,215.4	13,916.3
Vulcan BN Geothermal Power Co	006	P10	10,175.0	12,522.1
Vulcan BN Geothermal Power Co	007	P90	13,297.5	16,706.8
Vulcan BN Geothermal Power Co	007	P50	10,033.0	12,331.7
Vulcan BN Geothermal Power Co	007	P10	8,999.6	10,946.7
Vulcan BN Geothermal Power Co	008	P90	13,404.1	16,849.6
Vulcan BN Geothermal Power Co	008	P50	10,138.8	12,473.5
Vulcan BN Geothermal Power Co	008	P10	9,128.8	11,119.9
Region 1 Salton Sea Power Gener	006	P90	15,567.3	19,749.8
Region 1 Salton Sea Power Gener	006	P50	13,791.6	17,370.0
Region 1 Salton Sea Power Gener	006	P10	12,650.6	15,840.7
Region 1 Salton Sea Power Gener	007	P90	13,274.2	16,676.5
Region 1 Salton Sea Power Gener	007	P50	11,833.0	14,744.8
Region 1 Salton Sea Power Gener	007	P10	10,909.6	13,507.2
Region 1 Salton Sea Power Gener	008	P90	15,709.8	19,940.9
Region 1 Salton Sea Power Gener	008	P50	13,886.1	17,496.6
Region 1 Salton Sea Power Gener	008	P10	12,656.7	15,848.9
CTR's Hell's Kitchen	006	P90	14,220.9	17,856.7
CTR's Hell's Kitchen	006	P50	13,051.4	16,298.7
CTR's Hell's Kitchen	006	P10	11,932.2	14,807.7
CTR's Hell's Kitchen	007	P90	14,030.1	17,602.4
CTR's Hell's Kitchen	007	P50	12,863.4	16,048.2
CTR's Hell's Kitchen	007	P10	11,792.5	14,621.6
CTR's Hell's Kitchen	008	P90	14,044.3	17,621.5
CTR's Hell's Kitchen	008	P50	12,906.7	16,105.9
CTR's Hell's Kitchen	008	P10	11,823.0	14,662.2

Supplementary Table 5: Net present values (NPVs) (in million USD) for each scenario under lithium prices of 10,000, 20,000 and 30,000 USD/t LCE.

Scenario				NPVs for set lithium prices (in million USD)					
				10,000 USD/t LCE		20,000 USD/t LCE		30,000 USD/t LCE	
**Well field property**	**Operation scenario**	**Confidence interval**	**Production process**	*Excl. by-products sales*	*Incl. by-product sales*	*Excl. by-products sales*	*Incl. by-product sales*	*Excl. by-products sales*	*Incl. by-product sales*
John L. Featherstone-Hudson Ran.	006	P90	Carbonation & Conversion	(834.0)	(503.5)	(117.6)	140.4	485.5	706.4
John L. Featherstone-Hudson Ran.	006	P90	Electrolysis	(1,226.1)	(875.5)	(480.4)	(191.2)	174.3	404.2
John L. Featherstone-Hudson Ran.	006	P50	Carbonation & Conversion	(765.8)	(397.7)	25.6	313.1	700.3	953.1
John L. Featherstone-Hudson Ran.	006	P50	Electrolysis	(1,156.8)	(767.8)	(319.5)	(6.8)	397.9	655.2
John L. Featherstone-Hudson Ran.	006	P10	Carbonation & Conversion	(696.5)	(284.7)	176.7	497.2	933.2	1,220.5
John L. Featherstone-Hudson Ran.	006	P10	Electrolysis	(1,082.0)	(651.8)	(152.4)	185.8	635.9	923.3
John L. Featherstone-Hudson Ran.	007	P90	Carbonation & Conversion	(853.3)	(522.5)	(140.6)	114.5	454.9	672.6
John L. Featherstone-Hudson Ran.	007	P90	Electrolysis	(1,250.8)	(902.8)	(508.2)	(221.9)	139.5	366.2
John L. Featherstone-Hudson Ran.	007	P50	Carbonation & Conversion	(790.5)	(423.5)	(6.6)	275.4	654.8	902.3
John L. Featherstone-Hudson Ran.	007	P50	Electrolysis	(1,187.7)	(800.3)	(357.6)	(49.4)	347.6	600.1
John L. Featherstone-Hudson Ran.	007	P10	Carbonation & Conversion	(724.9)	(318.8)	132.4	444.4	867.9	1,147.1
John L. Featherstone-Hudson Ran.	007	P10	Electrolysis	(1,119.5)	(692.8)	(203.5)	127.4	566.2	845.4
John L. Featherstone-Hudson Ran.	008	P90	Carbonation & Conversion	(821.6)	(484.3)	(91.0)	172.6	526.9	754.1
John L. Featherstone-Hudson Ran.	008	P90	Electrolysis	(1,213.9)	(856.2)	(451.6)	(157.2)	215.9	452.1
John L. Featherstone-Hudson Ran.	008	P50	Carbonation & Conversion	(757.2)	(383.1)	46.1	338.9	733.8	991.8
John L. Featherstone-Hudson Ran.	008	P50	Electrolysis	(1,148.5)	(753.3)	(296.9)	19.0	431.1	693.3
John L. Featherstone-Hudson Ran.	008	P10	Carbonation & Conversion	(690.8)	(273.1)	193.0	518.1	960.7	1,252.4
John L. Featherstone-Hudson Ran.	008	P10	Electrolysis	(1,076.3)	(640.7)	(136.1)	206.2	662.7	954.4
JM Leathers LP	006	P90	Carbonation & Conversion	(836.5)	(506.3)	(129.3)	119.4	452.8	667.5
JM Leathers LP	006	P90	Electrolysis	(1,233.3)	(888.9)	(493.9)	(211.2)	141.1	365.2
JM Leathers LP	006	P50	Carbonation & Conversion	(795.5)	(444.7)	(48.1)	216.7	573.0	805.2
JM Leathers LP	006	P50	Electrolysis	(1,190.2)	(821.6)	(400.8)	(105.7)	267.5	505.1
JM Leathers LP	006	P10	Carbonation & Conversion	(728.3)	(345.1)	83.0	376.0	772.2	1,033.3
JM Leathers LP	006	P10	Electrolysis	(1,119.1)	(715.4)	(251.9)	62.0	474.5	735.6
JM Leathers LP	007	P90	Carbonation & Conversion	(796.9)	(452.2)	(58.7)	205.5	562.3	792.6
JM Leathers LP	007	P90	Electrolysis	(1,190.5)	(827.4)	(413.1)	(120.1)	253.5	493.0
JM Leathers LP	007	P50	Carbonation & Conversion	(757.2)	(391.5)	22.3	304.3	685.2	933.6
JM Leathers LP	007	P50	Electrolysis	(1,149.6)	(763.4)	(320.6)	(15.7)	381.5	635.0
JM Leathers LP	007	P10	Carbonation & Conversion	(704.2)	(307.4)	134.6	441.2	856.3	1,130.0
JM Leathers LP	007	P10	Electrolysis	(1,092.2)	(675.6)	(197.6)	127.3	558.9	832.5
JM Leathers LP	008	P90	Carbonation & Conversion	(947.3)	(682.3)	(376.3)	(160.2)	112.4	282.5
JM Leathers LP	008	P90	Electrolysis	(1,347.8)	(1,081.2)	(759.2)	(530.4)	(223.2)	(39.1)
JM Leathers LP	008	P50	Carbonation & Conversion	(914.2)	(629.5)	(301.5)	(76.0)	213.9	394.0
JM Leathers LP	008	P50	Electrolysis	(1,313.2)	(1,023.7)	(680.4)	(434.6)	(112.8)	81.7
JM Leathers LP	008	P10	Carbonation & Conversion	(863.0)	(550.5)	(191.2)	49.7	364.8	566.3
JM Leathers LP	008	P10	Electrolysis	(1,259.3)	(934.7)	(561.4)	(290.5)	50.6	262.7
Del Ranch LP	006	P90	Carbonation & Conversion	(1,190.8)	(761.6)	(263.7)	65.1	490.8	766.4
Del Ranch LP	006	P90	Electrolysis	(1,741.3)	(1,297.1)	(783.3)	(407.6)	61.8	348.0
Del Ranch LP	006	P50	Carbonation & Conversion	(1,134.6)	(677.2)	(147.8)	197.9	655.0	954.4
Del Ranch LP	006	P50	Electrolysis	(1,682.1)	(1,201.5)	(653.8)	(255.7)	234.4	538.2
Del Ranch LP	006	P10	Carbonation & Conversion	(1,094.8)	(616.4)	(66.8)	293.4	774.0	1,090.8
Del Ranch LP	006	P10	Electrolysis	(1,640.6)	(1,136.2)	(561.2)	(151.0)	359.0	675.8
Del Ranch LP	007	P90	Carbonation & Conversion	(1,015.9)	(505.9)	73.8	470.6	1,006.8	1,357.2
Del Ranch LP	007	P90	Electrolysis	(1,555.2)	(1,016.3)	(396.1)	30.5	589.5	945.8
Del Ranch LP	007	P50	Carbonation & Conversion	(949.5)	(402.1)	212.5	639.7	1,218.1	1,599.3
Del Ranch LP	007	P50	Electrolysis	(1,480.2)	(907.0)	(244.7)	207.3	809.2	1,190.4
Del Ranch LP	007	P10	Carbonation & Conversion	(899.5)	(320.0)	320.8	771.9	1,388.3	1,794.6
Del Ranch LP	007	P10	Electrolysis	(1,423.2)	(821.4)	(125.4)	346.5	980.4	1,386.7
Del Ranch LP	008	P90	Carbonation & Conversion	(989.4)	(477.5)	109.4	511.5	1,055.2	1,411.2
Del Ranch LP	008	P90	Electrolysis	(1,520.0)	(980.2)	(353.4)	78.1	644.4	1,005.1
Del Ranch LP	008	P50	Carbonation & Conversion	(919.2)	(363.7)	261.9	698.2	1,290.4	1,680.7
Del Ranch LP	008	P50	Electrolysis	(1,438.1)	(860.8)	(187.2)	273.2	886.7	1,277.0
Del Ranch LP	008	P10	Carbonation & Conversion	(866.3)	(275.2)	379.3	841.7	1,475.0	1,892.5
Del Ranch LP	008	P10	Electrolysis	(1,375.7)	(768.3)	(58.9)	422.9	1,072.7	1,490.2
JJ Elmore LP	006	P90	Carbonation & Conversion	(797.8)	(460.8)	(59.9)	213.9	579.9	814.0
JJ Elmore LP	006	P90	Electrolysis	(1,177.5)	(819.3)	(409.7)	(109.2)	277.0	520.3
JJ Elmore LP	006	P50	Carbonation & Conversion	(720.4)	(335.1)	113.9	423.5	841.1	1,114.0
JJ Elmore LP	006	P50	Electrolysis	(1,093.2)	(690.8)	(217.1)	112.9	548.0	825.1
JJ Elmore LP	006	P10	Carbonation & Conversion	(662.0)	(235.9)	249.1	588.5	1,049.0	1,352.5
JJ Elmore LP	006	P10	Electrolysis	(1,024.7)	(588.6)	(71.8)	284.1	761.1	1,064.6
JJ Elmore LP	007	P90	Carbonation & Conversion	(799.9)	(450.1)	(42.6)	235.3	609.7	849.7
JJ Elmore LP	007	P90	Electrolysis	(1,185.4)	(814.5)	(391.8)	(88.7)	303.2	552.0
JJ Elmore LP	007	P50	Carbonation & Conversion	(714.6)	(315.3)	141.1	457.8	887.7	1,168.8
JJ Elmore LP	007	P50	Electrolysis	(1,093.2)	(675.3)	(189.8)	145.9	591.4	876.4
JJ Elmore LP	007	P10	Carbonation & Conversion	(655.3)	(215.7)	275.2	622.3	1,095.2	1,406.8
JJ Elmore LP	007	P10	Electrolysis	(1,024.1)	(572.5)	(45.5)	316.8	803.9	1,115.6
JJ Elmore LP	008	P90	Carbonation & Conversion	(871.6)	(561.4)	(198.0)	51.8	378.1	583.5

JJ Elmore LP	008	P90	Electrolysis	(1,257.0)	(927.4)	(560.2)	(288.0)	65.9	283.2
JJ Elmore LP	008	P50	Carbonation & Conversion	(778.1)	(420.5)	(0.7)	282.2	662.2	909.3
JJ Elmore LP	008	P50	Electrolysis	(1,160.8)	(782.7)	(346.9)	(33.3)	364.3	617.6
JJ Elmore LP	008	P10	Carbonation & Conversion	(718.9)	(326.3)	126.7	437.8	856.3	1,132.1
JJ Elmore LP	008	P10	Electrolysis	(1,095.8)	(685.4)	(203.1)	128.2	565.7	841.5
Vulcan BN Geothermal Power Co	006	P90	Carbonation & Conversion	(237.7)	(169.6)	(91.5)	(33.7)	39.2	85.0
Vulcan BN Geothermal Power Co	006	P90	Electrolysis	(340.2)	(269.3)	(188.2)	(129.4)	(48.1)	1.3
Vulcan BN Geothermal Power Co	006	P50	Carbonation & Conversion	(191.5)	(99.3)	9.8	83.8	183.9	248.8
Vulcan BN Geothermal Power Co	006	P50	Electrolysis	(291.5)	(194.3)	(80.9)	0.9	105.0	172.0
Vulcan BN Geothermal Power Co	006	P10	Carbonation & Conversion	(175.0)	(71.4)	48.0	130.7	242.6	316.2
Vulcan BN Geothermal Power Co	006	P10	Electrolysis	(271.9)	(165.3)	(38.2)	49.3	165.8	239.7
Vulcan BN Geothermal Power Co	007	P90	Carbonation & Conversion	(218.3)	(141.7)	(51.1)	12.4	96.0	150.0
Vulcan BN Geothermal Power Co	007	P90	Electrolysis	(319.6)	(237.3)	(145.6)	(77.7)	12.6	68.2
Vulcan BN Geothermal Power Co	007	P50	Carbonation & Conversion	(172.7)	(68.1)	52.1	136.4	251.3	326.1
Vulcan BN Geothermal Power Co	007	P50	Electrolysis	(268.8)	(161.5)	(34.3)	54.7	173.3	249.8
Vulcan BN Geothermal Power Co	007	P10	Carbonation & Conversion	(150.8)	(31.9)	102.6	198.9	330.0	416.4
Vulcan BN Geothermal Power Co	007	P10	Electrolysis	(244.3)	(123.6)	19.4	119.1	254.2	340.6
Vulcan BN Geothermal Power Co	008	P90	Carbonation & Conversion	(219.6)	(144.1)	(54.7)	8.1	90.4	143.5
Vulcan BN Geothermal Power Co	008	P90	Electrolysis	(320.7)	(239.4)	(149.1)	(82.2)	7.1	61.8
Vulcan BN Geothermal Power Co	008	P50	Carbonation & Conversion	(175.1)	(72.3)	46.1	128.8	241.5	314.9
Vulcan BN Geothermal Power Co	008	P50	Electrolysis	(271.5)	(165.7)	(40.2)	47.2	163.5	238.7
Vulcan BN Geothermal Power Co	008	P10	Carbonation & Conversion	(154.3)	(37.6)	94.1	188.0	316.0	400.4
Vulcan BN Geothermal Power Co	008	P10	Electrolysis	(247.8)	(129.7)	10.6	108.3	240.3	324.6
Region 1 Salton Sea Power Gener	006	P90	Carbonation & Conversion	(2,514.3)	(1,757.8)	(889.0)	(262.0)	526.4	1,024.3
Region 1 Salton Sea Power Gener	006	P90	Electrolysis	(3,617.1)	(2,835.3)	(1,931.8)	(1,277.9)	(389.2)	140.6
Region 1 Salton Sea Power Gener	006	P50	Carbonation & Conversion	(2,345.9)	(1,493.9)	(493.3)	180.9	1,067.8	1,631.3
Region 1 Salton Sea Power Gener	006	P50	Electrolysis	(3,446.2)	(2,541.5)	(1,525.6)	(774.1)	187.0	781.0
Region 1 Salton Sea Power Gener	006	P10	Carbonation & Conversion	(2,210.3)	(1,291.4)	(211.4)	509.6	1,472.6	2,095.2
Region 1 Salton Sea Power Gener	006	P10	Electrolysis	(3,304.6)	(2,327.5)	(1,217.2)	(404.1)	615.4	1,262.5
Region 1 Salton Sea Power Gener	007	P90	Carbonation & Conversion	(2,294.9)	(1,437.1)	(427.5)	261.8	1,177.7	1,758.4
Region 1 Salton Sea Power Gener	007	P90	Electrolysis	(3,385.3)	(2,468.9)	(1,441.0)	(682.2)	295.3	903.3
Region 1 Salton Sea Power Gener	007	P50	Carbonation & Conversion	(2,097.0)	(1,127.7)	4.8	771.7	1,809.4	2,481.0
Region 1 Salton Sea Power Gener	007	P50	Electrolysis	(3,183.8)	(2,155.0)	(970.0)	(123.8)	956.6	1,652.7
Region 1 Salton Sea Power Gener	007	P10	Carbonation & Conversion	(1,951.5)	(894.0)	321.6	1,159.5	2,294.1	3,036.8
Region 1 Salton Sea Power Gener	007	P10	Electrolysis	(3,019.6)	(1,911.5)	(614.9)	279.6	1,459.6	2,212.3
Region 1 Salton Sea Power Gener	008	P90	Carbonation & Conversion	(2,527.2)	(1,785.2)	(934.7)	(317.6)	462.7	954.8
Region 1 Salton Sea Power Gener	008	P90	Electrolysis	(3,626.5)	(2,860.4)	(1,974.4)	(1,333.9)	(459.7)	64.3
Region 1 Salton Sea Power Gener	008	P50	Carbonation & Conversion	(2,359.1)	(1,522.1)	(538.2)	128.9	1,007.6	1,561.2
Region 1 Salton Sea Power Gener	008	P50	Electrolysis	(3,456.0)	(2,566.0)	(1,569.5)	(831.5)	120.9	709.0
Region 1 Salton Sea Power Gener	008	P10	Carbonation & Conversion	(2,215.7)	(1,308.3)	(238.9)	479.4	1,435.7	2,051.9
Region 1 Salton Sea Power Gener	008	P10	Electrolysis	(3,306.1)	(2,340.2)	(1,243.4)	(437.9)	577.5	1,219.3
CTR's Hell's Kitchen	006	P90	Carbonation & Conversion	(959.8)	(613.6)	(210.2)	60.2	408.0	632.7
CTR's Hell's Kitchen	006	P90	Electrolysis	(1,400.5)	(1,038.4)	(625.5)	(320.8)	63.3	297.5
CTR's Hell's Kitchen	006	P50	Carbonation & Conversion	(902.8)	(519.2)	(77.2)	213.0	597.1	850.3
CTR's Hell's Kitchen	006	P50	Electrolysis	(1,344.5)	(939.5)	(479.8)	(150.1)	261.1	514.3
CTR's Hell's Kitchen	006	P10	Carbonation & Conversion	(835.9)	(413.5)	61.7	375.8	807.2	1,091.6
CTR's Hell's Kitchen	006	P10	Electrolysis	(1,276.8)	(832.8)	(319.2)	29.4	471.9	756.2
CTR's Hell's Kitchen	007	P90	Carbonation & Conversion	(951.6)	(601.0)	(192.1)	80.6	434.1	662.6
CTR's Hell's Kitchen	007	P90	Electrolysis	(1,392.2)	(1,023.8)	(606.1)	(297.4)	89.9	327.5
CTR's Hell's Kitchen	007	P50	Carbonation & Conversion	(892.9)	(504.5)	(57.5)	236.1	626.9	884.4
CTR's Hell's Kitchen	007	P50	Electrolysis	(1,334.2)	(924.3)	(457.3)	(124.4)	291.2	548.7
CTR's Hell's Kitchen	007	P10	Carbonation & Conversion	(826.6)	(399.4)	79.8	398.3	836.2	1,124.8
CTR's Hell's Kitchen	007	P10	Electrolysis	(1,267.2)	(818.4)	(298.3)	52.4	501.0	789.7
CTR's Hell's Kitchen	008	P90	Carbonation & Conversion	(952.8)	(604.0)	(197.2)	74.7	426.2	653.4
CTR's Hell's Kitchen	008	P90	Electrolysis	(1,392.6)	(1,026.4)	(610.9)	(303.7)	82.5	318.7
CTR's Hell's Kitchen	008	P50	Carbonation & Conversion	(895.7)	(509.5)	(64.6)	227.6	615.7	871.5
CTR's Hell's Kitchen	008	P50	Electrolysis	(1,336.6)	(928.9)	(465.1)	(133.5)	280.3	536.1
CTR's Hell's Kitchen	008	P10	Carbonation & Conversion	(829.2)	(404.1)	73.5	390.3	825.7	1,112.6
CTR's Hell's Kitchen	008	P10	Electrolysis	(1,269.5)	(822.8)	(305.3)	44.5	490.8	777.7

Supplementary Table 6: Break-even (NPV=0) lithium prices (USD/t LCE) for each scenario.

**Scenario**				**Break-even (NPV=0) lithium prices (USD/t LCE)**	
**Well field property**	**Operation scenario**	**Confidence interval**	**Production process**	*Excl. by- products sales*	*Incl. by-product sales*
John L. Featherstone-Hudson Ran.	006	P90	Carbonation & Conversion	21,871.1	17,639.1
John L. Featherstone-Hudson Ran.	006	P90	Electrolysis	27,161.7	23,098.5
John L. Featherstone-Hudson Ran.	006	P50	Carbonation & Conversion	19,637.4	15,334.1
John L. Featherstone-Hudson Ran.	006	P50	Electrolysis	24,254.6	20,098.7
John L. Featherstone-Hudson Ran.	006	P10	Carbonation & Conversion	17,783.5	13,421.1
John L. Featherstone-Hudson Ran.	006	P10	Electrolysis	21,841.9	17,608.9
John L. Featherstone-Hudson Ran.	007	P90	Carbonation & Conversion	22,265.5	18,046.0
John L. Featherstone-Hudson Ran.	007	P90	Electrolysis	27,696.7	23,650.6
John L. Featherstone-Hudson Ran.	007	P50	Carbonation & Conversion	20,095.3	15,806.6
John L. Featherstone-Hudson Ran.	007	P50	Electrolysis	24,871.5	20,735.2
John L. Featherstone-Hudson Ran.	007	P10	Carbonation & Conversion	18,295.5	13,949.4
John L. Featherstone-Hudson Ran.	007	P10	Electrolysis	22,527.2	18,316.1
John L. Featherstone-Hudson Ran.	008	P90	Carbonation & Conversion	21,416.1	17,169.6
John L. Featherstone-Hudson Ran.	008	P90	Electrolysis	26,570.2	22,488.2
John L. Featherstone-Hudson Ran.	008	P50	Carbonation & Conversion	19,359.4	15,047.2
John L. Featherstone-Hudson Ran.	008	P50	Electrolysis	23,895.3	19,727.8
John L. Featherstone-Hudson Ran.	008	P10	Carbonation & Conversion	17,611.1	13,243.1
John L. Featherstone-Hudson Ran.	008	P10	Electrolysis	21,620.5	17,380.4
JM Leathers LP	006	P90	Carbonation & Conversion	22,122.6	17,898.4
JM Leathers LP	006	P90	Electrolysis	27,595.0	23,545.6
JM Leathers LP	006	P50	Carbonation & Conversion	20,736.3	16,468.1
JM Leathers LP	006	P50	Electrolysis	25,776.2	21,668.8
JM Leathers LP	006	P10	Carbonation & Conversion	18,859.0	14,530.8
JM Leathers LP	006	P10	Electrolysis	23,312.7	19,126.7
JM Leathers LP	007	P90	Carbonation & Conversion	20,910.1	16,647.4
JM Leathers LP	007	P90	Electrolysis	25,995.7	21,895.3
JM Leathers LP	007	P50	Carbonation & Conversion	19,678.9	15,376.9
JM Leathers LP	007	P50	Electrolysis	24,385.1	20,233.3
JM Leathers LP	007	P10	Carbonation & Conversion	18,232.9	13,884.7
JM Leathers LP	007	P10	Electrolysis	22,493.0	18,280.9
JM Leathers LP	008	P90	Carbonation & Conversion	27,536.3	23,485.1
JM Leathers LP	008	P90	Electrolysis	34,687.0	30,864.0
JM Leathers LP	008	P50	Carbonation & Conversion	25,658.8	21,547.7
JM Leathers LP	008	P50	Electrolysis	32,225.0	28,323.4
JM Leathers LP	008	P10	Carbonation & Conversion	23,283.4	19,096.4
JM Leathers LP	008	P10	Electrolysis	29,108.9	25,107.9
Del Ranch LP	006	P90	Carbonation & Conversion	23,293.1	19,106.5
Del Ranch LP	006	P90	Electrolysis	29,178.2	25,179.4
Del Ranch LP	006	P50	Carbonation & Conversion	21,734.4	17,498.1
Del Ranch LP	006	P50	Electrolysis	27,127.9	23,063.6
Del Ranch LP	006	P10	Carbonation & Conversion	20,751.3	16,481.5
Del Ranch LP	006	P10	Electrolysis	25,835.9	21,729.6
Del Ranch LP	007	P90	Carbonation & Conversion	19,245.8	14,930.0
Del Ranch LP	007	P90	Electrolysis	23,847.4	19,678.4
Del Ranch LP	007	P50	Carbonation & Conversion	17,998.4	13,642.8
Del Ranch LP	007	P50	Electrolysis	22,208.3	17,987.1
Del Ranch LP	007	P10	Carbonation & Conversion	17,134.4	12,752.0
Del Ranch LP	007	P10	Electrolysis	21,076.1	16,819.7
Del Ranch LP	008	P90	Carbonation & Conversion	18,898.0	14,571.1
Del Ranch LP	008	P90	Electrolysis	23,372.6	19,188.5
Del Ranch LP	008	P50	Carbonation & Conversion	17,580.5	13,212.4
Del Ranch LP	008	P50	Electrolysis	21,644.0	17,406.2
Del Ranch LP	008	P10	Carbonation & Conversion	16,702.1	12,304.6
Del Ranch LP	008	P10	Electrolysis	20,489.2	16,216.3
JJ Elmore LP	006	P90	Carbonation & Conversion	20,876.0	16,611.9
JJ Elmore LP	006	P90	Electrolysis	25,747.5	21,639.5
JJ Elmore LP	006	P50	Carbonation & Conversion	18,503.0	14,167.3
JJ Elmore LP	006	P50	Electrolysis	22,681.8	18,472.3
JJ Elmore LP	006	P10	Carbonation & Conversion	17,057.3	12,671.5
JJ Elmore LP	006	P10	Electrolysis	20,804.2	16,538.4
JJ Elmore LP	007	P90	Carbonation & Conversion	20,618.6	16,345.8
JJ Elmore LP	007	P90	Electrolysis	25,432.3	21,315.5
JJ Elmore LP	007	P50	Carbonation & Conversion	18,202.5	13,853.4
JJ Elmore LP	007	P50	Electrolysis	22,303.3	18,085.0
JJ Elmore LP	007	P10	Carbonation & Conversion	16,816.5	12,423.6
JJ Elmore LP	007	P10	Electrolysis	20,506.0	16,230.4
JJ Elmore LP	008	P90	Carbonation & Conversion	23,258.5	19,070.8
JJ Elmore LP	008	P90	Electrolysis	28,854.3	24,845.1
JJ Elmore LP	008	P50	Carbonation & Conversion	20,010.2	15,718.8
JJ Elmore LP	008	P50	Electrolysis	24,641.2	20,497.5
JJ Elmore LP	008	P10	Carbonation & Conversion	18,353.0	14,007.6
JJ Elmore LP	008	P10	Electrolysis	22,491.3	18,279.1

Vulcan BN Geothermal Power Co	006	P90	Carbonation & Conversion	26,806.3	22,731.8
Vulcan BN Geothermal Power Co	006	P90	Electrolysis	33,747.0	29,894.0
Vulcan BN Geothermal Power Co	006	P50	Carbonation & Conversion	19,460.1	15,152.8
Vulcan BN Geothermal Power Co	006	P50	Electrolysis	24,114.0	19,952.2
Vulcan BN Geothermal Power Co	006	P10	Carbonation & Conversion	17,663.5	13,296.0
Vulcan BN Geothermal Power Co	006	P10	Electrolysis	21,756.8	17,518.8
Vulcan BN Geothermal Power Co	007	P90	Carbonation & Conversion	23,315.1	19,127.9
Vulcan BN Geothermal Power Co	007	P90	Electrolysis	29,150.8	25,151.1
Vulcan BN Geothermal Power Co	007	P50	Carbonation & Conversion	17,520.6	13,149.7
Vulcan BN Geothermal Power Co	007	P50	Electrolysis	21,566.5	17,324.7
Vulcan BN Geothermal Power Co	007	P10	Carbonation & Conversion	15,735.8	11,307.9
Vulcan BN Geothermal Power Co	007	P10	Electrolysis	19,227.6	14,911.2
Vulcan BN Geothermal Power Co	008	P90	Carbonation & Conversion	23,595.0	19,417.3
Vulcan BN Geothermal Power Co	008	P90	Electrolysis	29,512.6	25,524.7
Vulcan BN Geothermal Power Co	008	P50	Carbonation & Conversion	17,757.5	13,393.2
Vulcan BN Geothermal Power Co	008	P50	Electrolysis	21,873.1	17,642.1
Vulcan BN Geothermal Power Co	008	P10	Carbonation & Conversion	15,996.4	11,576.9
Vulcan BN Geothermal Power Co	008	P10	Electrolysis	19,567.2	15,261.7
Region 1 Salton Sea Power Gener	006	P90	Carbonation & Conversion	26,067.2	21,969.1
Region 1 Salton Sea Power Gener	006	P90	Electrolysis	32,818.2	28,935.5
Region 1 Salton Sea Power Gener	006	P50	Carbonation & Conversion	23,015.1	18,819.6
Region 1 Salton Sea Power Gener	006	P50	Electrolysis	28,817.6	24,807.2
Region 1 Salton Sea Power Gener	006	P10	Carbonation & Conversion	21,202.4	16,949.0
Region 1 Salton Sea Power Gener	006	P10	Electrolysis	26,434.0	22,347.6
Region 1 Salton Sea Power Gener	007	P90	Carbonation & Conversion	22,535.4	18,324.5
Region 1 Salton Sea Power Gener	007	P90	Electrolysis	28,168.0	24,137.0
Region 1 Salton Sea Power Gener	007	P50	Carbonation & Conversion	19,974.3	15,681.6
Region 1 Salton Sea Power Gener	007	P50	Electrolysis	24,815.1	20,677.1
Region 1 Salton Sea Power Gener	007	P10	Carbonation & Conversion	18,445.4	14,103.5
Region 1 Salton Sea Power Gener	007	P10	Electrolysis	22,809.7	18,605.0
Region 1 Salton Sea Power Gener	008	P90	Carbonation & Conversion	26,493.5	22,409.0
Region 1 Salton Sea Power Gener	008	P90	Electrolysis	33,367.7	29,502.6
Region 1 Salton Sea Power Gener	008	P50	Carbonation & Conversion	23,328.7	19,143.2
Region 1 Salton Sea Power Gener	008	P50	Electrolysis	29,221.0	25,223.5
Region 1 Salton Sea Power Gener	008	P10	Carbonation & Conversion	21,364.0	17,115.8
Region 1 Salton Sea Power Gener	008	P10	Electrolysis	26,638.2	22,558.3
CTR's Hell's Kitchen	006	P90	Carbonation & Conversion	23,181.4	18,989.6
CTR's Hell's Kitchen	006	P90	Electrolysis	28,970.0	24,964.6
CTR's Hell's Kitchen	006	P50	Carbonation & Conversion	21,061.3	16,806.4
CTR's Hell's Kitchen	006	P50	Electrolysis	26,210.4	22,115.0
CTR's Hell's Kitchen	006	P10	Carbonation & Conversion	19,211.4	14,891.8
CTR's Hell's Kitchen	006	P10	Electrolysis	23,787.3	19,615.0
CTR's Hell's Kitchen	007	P90	Carbonation & Conversion	22,869.1	18,669.4
CTR's Hell's Kitchen	007	P90	Electrolysis	28,560.0	24,540.8
CTR's Hell's Kitchen	007	P50	Carbonation & Conversion	20,780.3	16,508.6
CTR's Hell's Kitchen	007	P50	Electrolysis	25,836.4	21,732.5
CTR's Hell's Kitchen	007	P10	Carbonation & Conversion	18,989.8	14,669.3
CTR's Hell's Kitchen	007	P10	Electrolysis	23,499.2	19,320.7
CTR's Hell's Kitchen	008	P90	Carbonation & Conversion	22,954.7	18,761.2
CTR's Hell's Kitchen	008	P90	Electrolysis	28,675.2	24,659.9
CTR's Hell's Kitchen	008	P50	Carbonation & Conversion	20,880.3	16,617.0
CTR's Hell's Kitchen	008	P50	Electrolysis	25,965.4	21,866.7
CTR's Hell's Kitchen	008	P10	Carbonation & Conversion	19,065.5	14,746.5
CTR's Hell's Kitchen	008	P10	Electrolysis	23,597.1	19,418.3

Supplementary Table 7: Details on considered lithium production projects from brines in USA, Canada, and Germany for large-scale process and costs modelling in this study.

**Project no.**		**1**	**2**	**3**	**4**	**5**
Project name		South West Arkansas Project	Upper Rhine Valley Project	Clearwater Project	Boardwalk Project	Kingersley Project
Company		Standard Lithium	Vulcan Energy	E3 Lithium	LithiumBank	Grounded Lithium
Location		USA	Germany	Canada	Canada	Canada
Data source (company publication)		Pre-Feasibility Study[1]	Definitive Feasibility Study[2]	Pre-Feasibility Study[3]	Preliminary Economic Assessment[4]	Preliminary Economic Assessment[5]
Publication date		18-Sep-23	13-Feb-23	20-Jun-24	22-Feb-24	30-Jun-23
Brine concentration	*ppm*	437	181.1	75.5	70.1	74
Life of mine (LOM)	*Year*	20	20	50	20	20
Brine throughput	*m^3/d*	43,200	77,760	232,500	250,000	72,384
LCE supply capacity	*t/a*	26,400	18,183	22,748	29,476	9,680
	t LOM	528,000	363,651	1,137,400	589,513	193,600
Process	*Extraction process*	DLE – selective sorption	DLE – selective sorption	DLE – selective sorption	DLE – selective sorption	DLE – selective sorption
	Li-salt production	Electrolysis	Electrolysis	Carbonation & Conversion	Electrolysis	Carbonation & Conversion
Total facility LOM costs (USD)	*CapEx*	1,273,975,126	1,496,000,000	2,465,476,000	2,159,700,000	335,492,000
	OpEx	2,865,763,860	2,236,000,000	9,370,200,000	3,104,000,000	857,820,000
	SusEx	248,721,000	-	1,771,339,000	131,000,000	-
	Closure	1,600,000	-	27,404,000	85,800,000	1,463,000
	Total	**4,390,059,986**	**3,732,000,000**	**13,634,419,000**	**5,480,500,000**	**1,194,775,000**

LCE production costs (USD/t LCE) excl. by-products	*CapEx*	2,412.8	4,113.8	2,167.6	3,663.5	1,732.9
	OpEx	5,427.6	6,148.7	8,238.3	5,265.4	4,430.9
	SusEx	471.1	-	1,557.4	222.2	-
	Closure	3.0	-	24.1	145.5	7.6
	Total	**8,314.5**	**10,262.6**	**11,987.4**	**9,296.7**	**6,171.4**

Supplementary Table 8: Average scale factors for modelling cost categories of large-scale lithium extraction from geothermal brines in the Salton Sea area in this study derived from average project cost categories of projects presented in Supplementary Table 7 above.

**Cost category**	**Expenditure**	**Scale reference (scale factor unit)**	**Values for scale factors (average of company data)**		Note
			Carbonation & Conversion	Electrolysis	
CapEx	Well field	Brine throughput (USD/m^3 brine)	0.4773	0.4773	
	Pre-treatment	Brine throughput (USD/m^3 brine)	0.2425	0.2425	
	Li extraction	Brine throughput (USD/m^3 brine)	0.2908	0.2908	
	Purification	Brine throughput (USD/m^3 brine)	0.2927	0.2927	
	Li-salt production	Brine throughput (USD/m^3 brine)	0.0297	0.5498	
	Utilities, Reagents, Buildings, etc.	Brine throughput (USD/m^3 brine)	0.1936	0.1936	
	Installation	Brine throughput (USD/m^3 brine)	0.1191	0.1191	
	Contingency	Brine throughput (USD/m^3 brine)	0.2460	0.2460	
	Owner's costs	Brine throughput (USD/m^3 brine)	0.0814	0.0814	
SusEx	Subtotal	Brine throughput (USD/m^3 brine)	0.7042	0.8674	
Closure	Subtotal	Brine throughput (USD/m^3 brine)	0.0168	0.0168	
OpEx	Labor	Brine throughput (USD/m^3 brine)	0.4855	0.4855	
	Power	Brine throughput (USD/m^3 brine)	0.0995	0.3606	
	Calcium carbonate (pre-treatment)	Lithium production (USD/t LCE)	2,888.0	2,888.0	*Estimated based on patent process and stochiometric calculations*
	Calcium hydroxide (pre-treatment)	Lithium production (USD/t LCE)	376.7	376.7	*Estimated based on patent process and stochiometric calculations*
	Scale inhibitors	Lithium production (USD/t LCE)	5.8	5.8	
	Corrosion inhibitors	Lithium production (USD/t LCE)	5.5	5.5	
	Sodium hydroxide	Lithium production (USD/t LCE)	136.5	-	
	Hydrochloric acid	Lithium production (USD/t LCE)	89.2	-	
	Sodium carbonate	Lithium production (USD/t LCE)	308.1	246.3	
	Sodium metabisulfite	Lithium production (USD/t LCE)	-	70.2	
	Calcium hydroxide (production)	Lithium production (USD/t LCE)	129.1	2.7	
	Nitrogen	Lithium production (USD/t LCE)	-	14.5	
	Natural gas and water	Brine throughput (USD/m^3 brine)	0.0956	0.0108	
	Disposal	Lithium production (USD/t LCE)	322.8	322.8	
	Product transport	Lithium production (USD/t LCE)	196.8	196.8	
	Maintenance	Brine throughput (USD/m^3 brine)	0.2437	0.9787	
	G&A	Brine throughput (USD/m^3 brine)	0.1673	0.1673	
	Other	Brine throughput (USD/m^3 brine)	0.1405	0.1405	

Supplementary Table 9: Parameter applied for NPV calculations in this study for the Salton Sea region in California.

**Parameter**	**Value**	**Note**
Discount rate	7%	
U.S. federal tax rate	21%	
California state tax rate	8.84%	
Annual inflation rate	2.5%	
Royalty rate	3%	
Working capital recovery rate	90%	
Property tax rate	1.2%	
Depreciation rate schedule (MACRS)	14.29%; 24.49%; 17.49%; 12.49%; 8.93%; 8.92%; 8.93%; 4.46%	*For first seven years of mining*
Time of plant construction	3 years	
Production years (total; starting 2025)	30 years	
CapEx spending schedule	50%; 35%; 15%	*During three years plant construction*
California state lithium extraction excise tax	826 USD/t LCE	*Effective date: January 1, 2025; for over 30,000 t LCE extracted*

Supplementary Table 10: Parameter applied for by-product revenue calculation.

Parameter	Value	Note/source
Zinc hydroxide ($Zn(OH)_2$) selling price (over LOM)	2,200 USD/t	*Price equivalent to zinc oxide* https://www.metal.com/Zinc/201102250067
Manganese hydroxide ($Mn(OH)_2$) selling price (over LOM)	750 USD/t	*75% payable of metal manganese* https://www.imarcgroup.com/news/manganese-price-trend
Calcium chlorde ($CaCl_2$) selling price (over LOM)	300 USD/t	https://www.imarcgroup.com/calcium-chloride-pricing-report
Calcium ferro silicate ($Ca_3Fe_2Si_3O_{12}$) selling price (over LOM)	-	*Not sold; no market*
Revenue loss due to precipitation processes, efficiency rate, and transport costs (over LOM)	20%	*Conservative assumption*

Supplementary Note 1: Pre-treatment reagent cost calculations methodology

In this study, we modelled the pre-treatment process of the geothermal brines for each property in the Salton Sea region according to patented processes for the removal of major impurities in the brine by precipitation[6]. More specifically, the patented process establishes a 4-step method to precipitate iron, silicates, zinc, and manganese as main impurities to increase lithium extraction and production efficiency in the downstream process, as supplementary equations (1-5). The first step in this process is the oxidization of iron by adding calcium carbonate supplementary equation (1). Second, by further adding calcium carbonate, produced iron hydroxide reacts with hydro silicates to from calcium ferro silicates that precipitate, supplementary equation (2). Supplementary equation (3) represents the net chemical reaction for removing iron and silica. Finally, manganese and zinc are subsequently removed from the brine by precipitation after adding calcium hydroxide, supplementary equations (4) and (5)[6].

Iron Oxidation

$$2CaCO_3 + 2Fe^{2+} + 3H_2O + 0.5O_2 \rightarrow 2Fe(OH)_3 + 2CO_2 + 2Ca^{2+} \quad (1)$$

Iron and Silicate Precipitation

$$3CaCO_3 + 3H_4SiO_4 + 2Fe(OH)_3 \rightarrow Ca_3Fe_2Si_3O_{12} + 3CO_2 + 9H_2O \quad (2)$$

Net Reaction for Iron and Silica

$$5CaCO_3 + 2Fe^{2+} + 3H_4SiO_4 + 0.5O_2 \rightarrow Ca_3Fe_2Si_3O_{12} + 5CO_2 + 6H_2O + 2Ca^{2+} \quad (3)$$

Manganese

$$MnCl_2 + Ca(OH)_2 \rightarrow Mn(OH)_2(s) + CaCl_2 \quad (4)$$

Zinc

$$ZnCl_2 + Ca(OH)_2 \rightarrow Zn(OH)_2(s) + CaCl_2 \quad (5)$$

According to the stoichiometry of each chemical reaction presented above, we calculated the net reagents consumption of calcium carbonate and calcium hydroxide during the modelled pre-treatment process in this study. For this, we rely data for brine characterization data for impurities in the Salton Sea geothermal brines, which are based on a baseline lithium concentration of 202 ppm: Iron 1347 mg/L; silica 342 mg/L; zinc: 463 mg/L; manganese 1201 mg/L[7]. To remodel impurity content dilution over time, we based our approach on this data set and proportionately scaled impurity contents according to measured and modelled lithium content over time (see manuscript Table 1, Supplementary Figure 1, and Supplementary Tables 1-3) using 202 ppm of lithium as a proxy for impurity content calculations. For example, the P90/business-as-usual (006) scenario for the John L. Featherstone-Hudson Ranch property shows a starting lithium content of 174.07 ppm, which results in a modelled manganese concentration of (1201 mg/L * 174.07 ppm / 202 ppm =) 1,035 mg/L. Accounting for individual properties' total brine throughputs and assuming 100% removal of impurities during pre-treatment, we modelled total volumes of removed impurities and calculated required reagent consumptions based on individual compounds' molar masses and stoichiometry of each chemical reaction (Eq. 1 to Eq. 5). For calculating total pre-treatment reagent cost per scenario, we multiplied the total volume of calcium carbonate and calcium hydroxide over total life-of-mine (LOM) with market prices for both reagents that are assumed to remain constant over time (calcium carbonate: 400 USD/t; calcium hydroxide: 170 USD/t)[8, 9].

Supplementary Note 2: Calculation logic and methodology for projected domestic lithium demand

To estimate the domestic lithium demand, we first considered global lithium demand considering lithium for electric vehicles and energy storage systems. We utilized recently published global lithium demand models[10] which places 2025 global demand of LCE at ~1,250 kilo-tonnes (kt) of LCE. The US represents ~ 19% of global demand, resulting in a 2025 baseline demand of ~237.5 kt of LCE. The 19 % percentage was extrapolated from 2025 to 2035.

Supplementary References

1. Frank Gay, M. D., Randal M. Brush, Robert E. Williams, Caleb Mutschler, Dutch Johnson, Charles Campbell, Technical Report South West Arkansas Project Pre-Feasibility Study. **2023**.
2. GmbH, V. E. R. *Vulcan Zero Carbon Lithium Project Phase One DFS Results and Resources-Reserves Update*; 2023.
3. Daron Abbey, A. M. H., Meghan Klein, Antoine Lefaivre, Keith F. Wilson, *Clearwater Project NI 43-101 Technical Report on Pre-Feasibility Study*; E3 Lithium: 2024.
4. Hatch Ltd., G. L., APEX Geoscience Ltd., Scott Energy Inc., Go2Lithium *NI 43-101 Technical Report Preliminary Economic Assessment (PEA) FOR LithiumBank Resources Boardwalk Lithium-Brine Project in West-Central Alberta, Canada*; LithiumBank Resources Corp.: 2024.
5. Doug Ashton, A. R., Dean Quirk, Chad Hitchings, Michael Munteau, Jeffrey Weiss *NI 43-101 Technical Report: Preliminary Economic Assessment Kindersley Lithium Project-Phase 1 Update*; Grounded Lithium Corp.: 2023.
6. Featherstone, J. L.; Hanson, P. J.; Garska, M. J.; Marston, C. R., Process for recovery of lithium from a geothermal brine. Google Patents: 2025.
7. Dobson, P.; Araya, N.; Brounce, M.; Busse, M.; Camarillo, M. K.; English, L.; Humphreys, J.; Kalderon-Asael, B.; McKibben, M.; Millstein, D., Characterizing the geothermal lithium resource at the Salton Sea. **2023**. Lawrence Berkeley National Laboratory Report LBNL-2001557, 350 p.
8. Calcium hydroxide price index. https://businessanalytiq.com/procurementanalytics/index/calcium-hydroxide-price-index/.
9. Calcium carbonate price index. https://businessanalytiq.com/procurementanalytics/index/calcium-carbonate-price-index/.
10. Busch, P.; Chen, Y.; Ogbonna, P.; Kendall, A., Effects of demand and recycling on the when and where of lithium extraction. *Nat. Sustain.* **2025,** *8* (7), 773-783.
