# Supplementary material for: Pathways to cost competitive and viable lithium production from Salton Sea geothermal brines
Source: Nat Commun. 2026 Jul 9;17:8448. doi: 10.1038/s41467-026-75389-8 (PMC13478165; doi:10.1038/s41467-026-75389-8)
Supplement: Supplementary file 2 [file 41467_2026_75389_MOESM2_ESM.pdf]

nature portfolio

Peer Review File

Pathways to Cost Competitive and Viable Lithium Production from Salton Sea Geothermal Brines

Corresponding Author: Dr Andrew Haddad

This file contains all reviewer reports in order by version, followed by all author rebuttals in order by version.

Version 0:

Reviewer comments:

Reviewer #2

(Remarks to the Author)
In this manuscript, the authors conduct a facility-scale techno-economic assessment of LiOH production from Salton Sea geothermal brines, quantifying cost ranges across resource/operation scenarios and identifying key cost drivers and pathways to cost-competitiveness, such as by-product, CapEx support, and process optimization. The overall assessment and analysis are very solid, a few issues need to be addressed to further improve the quality of this manuscript:
1. The abstract is too general and too vague. The authors should include some key takeaways/findings of this work.
2. In Fig.2, the authors discussed the ion sieving adsorption as the DLE method. Considering the impurity ions are mainly K and Na, solvent extraction can also be a good DLE method in this scenario, and the cost might be even lower.
3. During the long-term operation, there is the aging of the adsorbents for ion sieving adsorption, and aging of CEM/AEM for electrodialysis. The authors should also consider these factors in the cost calculation.
4. In Supplementary Table 7, other than the concentration of brines, the impurity ratio, e.g., Li/Na or Li/Mg ratio, should be considered as a factor related to the extraction cost.
5. In Fig. 3h, the “power” cost contributes significantly to the overall cost of electrodialysis, while “power” cost is not shown in the carbonation & conversion process. However, the carbonation & conversion process still needs power input to stir the reactor and heat.
6. The caption of Fig. 3h is missing.

Reviewer #3

(Remarks to the Author)
Overall, this article is informative and well researched. With minor revisions, it should be suitable for publication.
Here are minor concerns that should be addressed:
1. Lines 164–175: The authors state that hard-rock production is the cheapest route. In most comparisons, brine-based production is the lowest-cost pathway, while clay and hard-rock are typically higher and often comparable (depending on project specifics and cost boundaries). Please revisit this statement, verify it against more literature, and support the revised claim with stronger, up-to-date references.
2. Lines 154–163: Please clarify why the P90 cost is higher for the JM Leathers LP location.
3. Line 180: Please add a description for (h) and explain why “CapEx for processing plants” (rather than “SusEx”) is selected for the sensitivity analysis. As written, this choice is unclear and potentially confusing to the reader.
4. The manuscript identifies three major cost contributors and includes a sensitivity analysis with suggested improvements. It would strengthen the paper to make the underlying assumptions for these three drivers more transparent. Reagent costs are relatively clear, but labor and CapEx assumptions are less explicit. For example, please explain how labor cost is calculated from Supplementary Table 7 to Supplementary Table 8, and how these labor assumptions relate to the model presented in Figure 2 (or clarify if they are independent).
5. Building on the earlier point about labor assumptions, improving traceability and reproducibility would strengthen the analysis. For example, please clarify how key assumptions differ between the carbonation & conversion pathway and electrolysis, particularly with respect to CapEx requirements (what are the assumptions that make electrolysis more capital expensive). Additionally, provide more detail on the assumptions driving the JM Leathers LP P90 case (and why it differs from other locations), so readers can reproduce/understand the outcome in figure 3 b.
6. Figure 5b: The assumed 50% OpEx reduction is substantial, but the manuscript does not provide concrete, defensible

pathways to achieve it beyond a general statement about process optimization and automation reducing labor and maintenance costs (line 201). It would be good to research feasible cost reduction for OpEx and implement that example in the discussion of figure 5b.

Version 1:

Reviewer comments:

Reviewer #3

(Remarks to the Author)
The revisions have adequately addressed my comments, and I am satisfied with the authors’ responses. In my view, the article is now suitable for release.

Open Access This Peer Review File is licensed under a Creative Commons Attribution 4.0 International License, which permits use, sharing, adaptation, distribution and reproduction in any medium or format, as long as you give appropriate credit to the original author(s) and the source, provide a link to the Creative Commons license, and indicate if changes were made.
In cases where reviewers are anonymous, credit should be given to 'Anonymous Referee' and the source.
The images or other third party material in this Peer Review File are included in the article’s Creative Commons license, unless indicated otherwise in a credit line to the material. If material is not included in the article’s Creative Commons license and your intended use is not permitted by statutory regulation or exceeds the permitted use, you will need to obtain permission directly from the copyright holder.
To view a copy of this license, visit https://creativecommons.org/licenses/by/4.0/

Response to reviewers' comments for:
Large-scale lithium production from geothermal brines at the Salton Sea: Pathways to cost-competitiveness and viability
Nature Communications

We would like to thank the Editor and the Reviewers for taking the time to review our manuscript and for providing constructive comments and suggestions that have greatly helped us further improving the paper. In the following we respond point-by-point to the comments and suggestions provided.

Reviewer #2:

In this manuscript, the authors conduct a facility-scale techno-economic assessment of LiOH production from Salton Sea geothermal brines, quantifying cost ranges across resource/operation scenarios and identifying key cost drivers and pathways to cost-competitiveness, such as by-product, CapEx support, and process optimization. The overall assessment and analysis are very solid, a few issues need to be addressed to further improve the quality of this manuscript:

Thank you for your positive feedback and recommendation to publish in Nature Communications. We appreciate your time and expertise. Your comments motivate us to continue advancing research in battery material supply cost and resilience. Thank you again.

Comments:

1. The abstract is too general and too vague. The authors should include some key takeaways/findings of this work.

We appreciate your feedback regarding the abstract and agree that it was initially too general. In response, we have revised the abstract to include specific key findings and takeaways from our facility-scale techno-economic assessment. These additions highlight the quantified cost ranges and outcomes of our project feasibility scenario analysis for lithium production from Salton Sea geothermal brines, ensuring the abstract more accurately reflects the main contributions of our work (see Lines 18 to 26 in revised manuscript).

2. In Fig.2, the authors discussed the ion sieving adsorption as the DLE method. Considering the impurity ions are mainly K and Na, solvent extraction can also be a good DLE method in this scenario, and the cost might be even lower.

Thank you for your valuable comment regarding the DLE methods considered here. We appreciate your suggestion considering solvent extraction as a potentially cost-effective DLE method, particularly given the dominant impurity ions in Salton Sea geothermal brines. We acknowledge that solvent extraction could offer advantages in this context and have expanded our discussion to reflect this possibility (sees Line 309 to 310 in revised manuscript). However, we would also like to highlight that a key strength and novelty of our study lie in leveraging benchmark primary cost data at the facility scale, rather than relying on uncertain up-scaling from laboratory or pilot data. By utilizing primary data from technologies with high technology readiness levels (TRL) such as those implemented in technical reports and feasibility studies by Vulcan Energy, Standard Lithium, and E3, we provide a more robust and realistic techno-economic assessment of large-scale lithium production. This approach ensures that our cost analysis is grounded in real-world operational data, enhancing the reliability and relevance of our findings.

3. During the long-term operation, there is the aging of the adsorbents for ion sieving adsorption, and aging of CEM/AEM for electrodialysis. The authors should also consider these factors in the cost calculation.

Thank you for raising the important issue of adsorbent and membrane aging during long-term operation. We agree that replacement and degradation of these materials should be considered in the cost analysis to ensure realistic estimates. Indeed, in our techno-economic assessment, we have incorporated adsorbent consumption and the periodic replacement of cation/anion exchange membranes (CEM/AEM) as part of the cost modelling. Specifically, these consumables and their expected lifetimes are included in the sustaining capital expenditures (SusEx). This approach

captures the costs associated with regular maintenance and material replacement, thereby reflecting the impact of aging on overall process economics.

4. In Supplementary Table 7, other than the concentration of brines, the impurity ratio, e.g., Li/Na or Li/Mg ratio, should be considered as a factor related to the extraction cost.

We appreciate this suggestion and agree that these ratios can significantly influence the efficiency and economics of lithium extraction from geothermal brines, in particular the pre-treatment and extraction/DLE phases. In our current techno-economic model, we assume that the DLE process, particularly when using Al-based adsorbents, is sufficiently selective to extract lithium from pre-treated brine with high efficiency. This assumption is based on the demonstrated selectivity of these materials in separating lithium ions from major impurities, which is a key reason for their industrial use, as expressed in the technical report summaries by Standard Lithium and other companies used as technology and cost benchmarks. However, we acknowledge that in practical applications, variations in impurity ratios can impact process performance and cost. To discuss this issue and method limitation, we highlight: "Certain cost inputs rely on analogues from other brine systems or geothermal regions, introducing uncertainty in transferability." However, we now explicitly call out the impact of impurity ratios within other brines (see Lines 288 to 289 in revised manuscript).

5. In Fig. 3h, the "power" cost contributes significantly to the overall cost of electrodialysis, while "power" cost is not shown in the carbonation & conversion process. However, the carbonation & conversion process still needs power input to stir the reactor and heat.

Thank you for your observation regarding Figure 3h in this and the next comment. Indeed, a caption of Figure 3h is missing, which eventually leads to a crucial misunderstanding you highlighted correctly. In this figure, we present only the eight most sensitive cost factors. The power requirement for the carbonation and conversion process contributes less to the overall cost per ton of LCE produced than for the electrolysis process, and therefore, it does not appear among the top eight factors in our sensitivity analysis. We added a caption to Figure 3h to clarify

6. The caption of Fig. 3h is missing.

Please refer to comment 5 above. We appreciate the comment and have added the necessary captions that now provide much needed information. Thank you.

Reviewer #3:

Overall, this article is informative and well researched. With minor revisions, it should be suitable for publication. Here are minor concerns that should be addressed:

Thank you very much for your positive feedback and thoughtful evaluation of our manuscript. We are grateful for your recognition of the article's informativeness and research quality, and we appreciate your constructive suggestions for minor revisions. Your comments have been invaluable in helping us strengthen the work, and we look forward to addressing the remaining points to ensure the manuscript meets the highest standards for publication.

Comments:

1. Lines 164–175: The authors state that hard-rock production is the cheapest route. In most comparisons, brine-based production is the lowest-cost pathway, while clay and hard-rock are typically higher and often comparable (depending on project specifics and cost boundaries). Please revisit this statement, verify it against more literature, and support the revised claim with stronger, up-to-date references.

We thank the reviewer for this important observation and for pushing us to verify this comparison against current literature. Upon revisiting recent market analyses, we agree that our original benchmarking did not accurately reflect the current cost landscape and have revised the text accordingly.

Recent industry and academic analyses indicate that, in the current market (2024–2025), high-grade continental brine operations remain the lowest-cost route, with total cash costs typically in the range of ~4,000–6,000 USD/t LCE (e.g., SQM and Albemarle operations at expanded salar sites). Hard-rock spodumene operations span a much wider cost range: top-tier integrated projects such as Greenbushes remain competitive (~4,000–6,000 USD/t LCE), but average Australian spodumene producers operate at ~12,000–15,000 USD/t LCE, and Chinese lepidolite-based production at ~8,000–10,000 USD/t LCE. Clay-hosted resources, which have matured significantly since earlier estimates, show more favorable economics than previously assumed: Lithium Americas' 2024 Technical Report for Thacker Pass reports C1 costs of ~6,238 USD/t LCE and AISC of ~7,508 USD/t LCE.

Accordingly, we have revised the passage (Lines 164–181) to reflect a more accurate and up-to-date picture:

'Setting these costs into context reveals significant gaps. Recent industry cost-curve analyses indicate that, in the current market, high-grade continental brines represent the lowest-cost production pathway (~4,000–6,000 USD/t LCE), while hard-rock spodumene operations span a wide range (~6,000–15,000 USD/t LCE) depending on deposit grade, scale, and degree of vertical integration, with top-tier integrated projects (e.g., Greenbushes) remaining competitive and average producers operating at the higher end. Clay-hosted resources, exemplified by the Thacker Pass project, have matured to cash costs/all-In Sustaining Costs (C1/AISC) estimates of ~6,200–7,500 USD/t LCE. Against these benchmarks, Salton Sea geothermal brines, given their lower lithium concentrations (~150–200 mg/L) compared with South American salars (~1,500–2,500 mg/L), face a substantial cost gap and require targeted optimization to become long-term cost-competitive.'

We have updated references to include the most recent Lithium Americas' 2024 NI 43-101 Technical Report for Thacker Pass, and recent peer-reviewed techno-economic comparisons (e.g., Gallagher et al., Advanced Energy Materials, 2025). We thank the reviewer again for prompting this correction, which strengthens the accuracy and relevance of our benchmarking."

2. Lines 154–163: Please clarify why the P90 cost is higher for the JM Leathers LP location.

Strong dilution of brine lithium concentration (in ppm) over time can significantly impact both the operational efficiency and cost structure of the extraction processes. As lithium concentrations decrease, more extensive processing and higher volumes of feedstock are required to maintain production levels, which in turn raises reagent use, labor input, and energy consumption. Based on the individual brine modelling of each property, dilution profiles

vary and, combined with unique impurity and plant throughput characteristics, result in individual large-scale production cost for each property considered in this study. Based on the results on reservoir modeling, the JM Leathers LP site is characterized by high dilution over time, high brine throughput and low lithium concentration, leading to higher cost compared to other locations.

3. Line 180: Please add a description for (h) and explain why "CapEx for processing plants" (rather than "SusEx") is selected for the sensitivity analysis. As written, this choice is unclear and potentially confusing to the reader.

Thanks for this observation. We added a caption to Figure 3h as requested. To address the concern regarding CapEx sensitivity, we want to clarify that all CapEx values are aggregated in accordance with standard industry practice, where capital expenditure grants and funding are typically allocated for the entire project rather than being tied to individual processing plants, such as DLE or electrolysis units. This approach ensures that the sensitivity analysis accurately reflects the project-wide financial structure and aligns with common funding mechanisms in large-scale industrial developments.

4. The manuscript identifies three major cost contributors and includes a sensitivity analysis with suggested improvements. It would strengthen the paper to make the underlying assumptions for these three drivers more transparent. Reagent costs are relatively clear, but labor and CapEx assumptions are less explicit. For example, please explain how labor cost is calculated from Supplementary Table 7 to Supplementary Table 8, and how these labor assumptions relate to the model presented in Figure 2 (or clarify if they are independent).

Thank you for your comment regarding the transparency of labor cost assumptions. To clarify, labor costs, as well as most cost drivers, are calculated using average scaling factors, which are derived from primary company data on lithium extraction costs of brine resources and reflect typical requirements for both labor and maintenance across similar projects. These costs are aggregated because labor and maintenance are closely related but distinct expense categories, each necessitating dedicated human resources. The methodologies for these scaling factors are described in detail in the methods and supporting information files.

5. Building on the earlier point about labor assumptions, improving traceability and reproducibility would strengthen the analysis. For example, please clarify how key assumptions differ between the carbonation & conversion pathway and electrolysis, particularly with respect to CapEx requirements (what are the assumptions that make electrolysis more capital expensive). Additionally, provide more detail on the assumptions driving the JM Leathers LP P90 case (and why it differs from other locations), so readers can reproduce/understand the outcome in figure 3 b.

We appreciate the feedback and would like to clarify that our CapEx estimates are calculated using scaling factors that are grounded in benchmark costs derived from primary company data for both electrolysis and carbonation/conversion processes (e.g., CapEx for electrolysis units and carbonation and conversion process plants). This methodology ensures that our cost assumptions are transparent and aligned with industry standards, as the scaling factors, which rely on CapEx spent per throughput volume (see methods and Supplementary Table 8) reflect actual project requirements and CapEx observed in comparable lithium extraction operations. By leveraging primary data, we strengthen the reproducibility and traceability of our analysis, providing a robust foundation for the sensitivity analysis and cost modeling presented in the manuscript.

6. Figure 5b: The assumed 50% OpEx reduction is substantial, but the manuscript does not provide concrete, defensible pathways to achieve it beyond a general statement about process optimization and automation reducing labor and maintenance costs (line 201). It would be good to research feasible cost reduction for OpEx and implement that example in the discussion of figure 5b

Indeed, we do not provide specific pathways to achieve substantial CapEx or OpEx (i.e., labor and maintenance) reductions. The core and value of our analysis is to demonstrate the effect of significant optimization and to highlight key cost drivers, such as pre-treatment reagent costs, and key levers to achieve feasibility, such as selling brine pre-treatment by-products, we acknowledge that identifying and implementing feasible cost reduction strategies extends beyond the intended scope of this study. Our focus is to illustrate the impact of these optimization measures on overall

project economics, and we encourage further research into practical pathways for achieving such reductions in operational expenditures.
